# Orthogonal Regioselective Synthesis of Folic Acid γ‑Conjugates

**DOI:** 10.1021/acs.orglett.5c04256

**Published:** 2025-11-27

**Authors:** Ciera F. Connelly, Shiao Y. Chow

**Affiliations:** Department of Pure and Applied Chemistry, 3527 University of Strathclyde, 295 Cathedral Street, Glasgow G1 1XL, Scotland

## Abstract

γ-Folic acid conjugates are valuable tools for targeting the folate receptor, an important cancer biomarker, but direct conjugation of folic acid is hampered by poor regioselectivity and challenging purification. We report an orthogonal protection strategy that enables controlled regioselective access to γ-folic acid conjugates from readily available building blocks. This approach permits site-selective installation of a range of functional modalities, including an amphiphilic ligand, a fluorescent probe, and a bioorthogonal handle, followed by global deprotection to afford γ-conjugates without the need for reversed phase chromatography.

## Graphical abstract

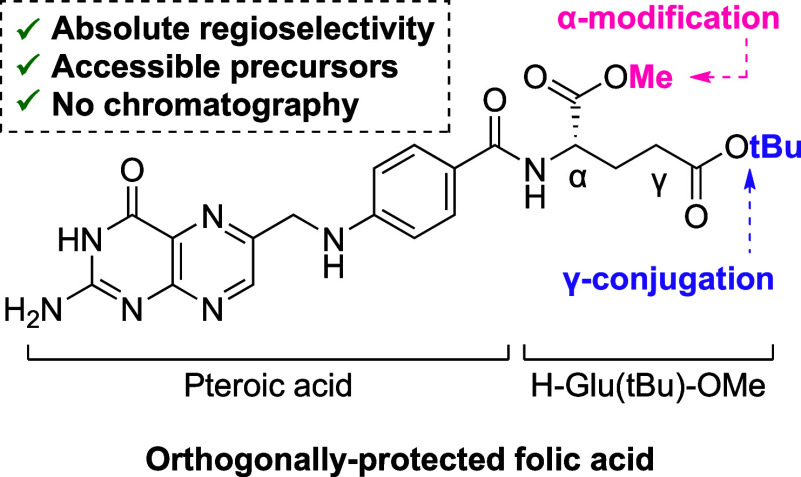

Folate receptor alpha (FRα) has emerged as a promising target for cancer therapy due to its pronounced overexpression in a variety of epithelial cancers. FRα plays a critical role in cell metabolism, DNA synthesis, and DNA repair in both normal and tumor cells.^1^ It was reported that overexpression of FRα promotes tumor growth through modulation of folate uptake and/or by inducing regulatory signals.

Folic acid is a high-affinity targeting ligand for FRα (*K*_d_ < 1 nM).^2^ Its efficient receptor-mediated internalization renders folic acid a valuable tumor-homing motif for targeted chemotherapeutic delivery. Folic acid features pteroic acid coupled to l-glutamic acid via an amide linkage (Figure 1A). The crystal structure revealed that the pteroate and α-carboxylic acid of the glutamic acid are critical to preserve high-affinity binding to the receptor, while the γ-carboxylic acid is solvent-exposed and thus represents an optimal site for vector growth (Figure 1B).^3^ Indeed, various reports of γ-folic acid conjugates armed with a variety of modalities for therapeutic delivery or diagnostic imaging applications have been reported.^4^

**Figure 1:**
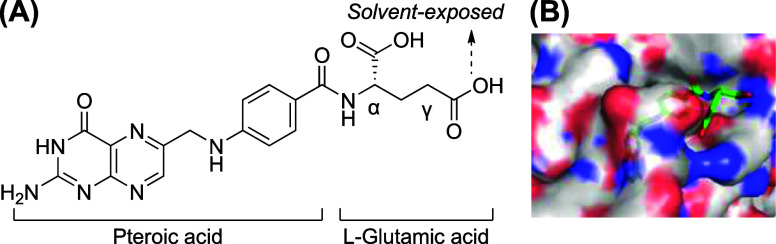
(A) Folic acid. (B) Co-crystal structure of folic acid with FRα (Protein Data Bank entry 4LRH).

Despite growing interest in folate receptor-targeted interrogation, efficient and regioselective access to γ-folate conjugates remains challenging. Most reported strategies have relied on the direct use of unmodified folic acid for γ-conjugation (Scheme 1A).^5^ However, the absence of orthogonal protection can often lead to nonselective attachment at the γ- and/or α-carboxyl sites. A recent study by Thompson and co-workers provided critical insight into the shortcomings of widely used direct coupling approaches, demonstrating that such reactions generated mixtures of regiosiomers, including γ- and α-conjugates.^6^ Despite incomplete characterization, these mixtures are frequently found in the literature with claimed receptor-targeted activity.

**Scheme 1:**
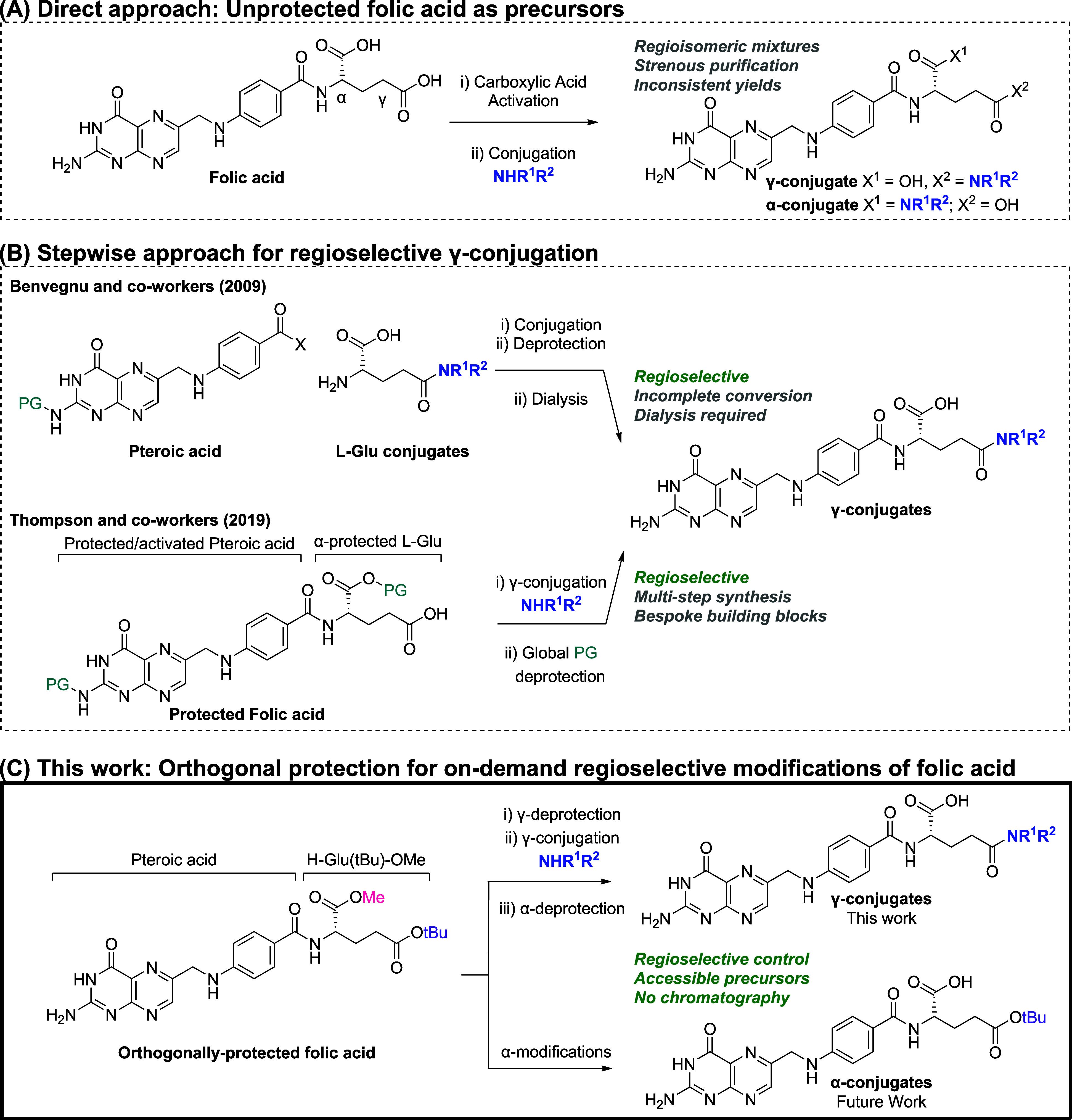
Synthesis Routes to Access Folic Acid γ-Conjugates

In addition, the isolation of pure γ-conjugates poses significant practical challenges. Folic acid derivatives are polar and sparingly soluble in most organic solvents, necessitating the use of DMSO (or DMF in certain cases) in the reactions. This inherent insolubility in common organic solvents also renders standard flash chromatography ineffective, instead requiring reverse phase purification. Together, strenuous purification requirements and reproducibility issues present substantial barriers to the efficient preparation of γ-conjugates.

To overcome these challenges, several groups have developed stepwise routes to achieve regioselective access to γ-folic acid conjugates from pteroic acid precursors. Two main approaches are reported: (1) coupling pteroic acid with preassembled γ-functionalized glutamic acid derivatives^7^ or (2) coupling *N*-protected pteroic acid^6,8^ with an α-protected glutamic acid, followed by site-selective γ-functionalization and subsequent global deprotection to yield the desired γ-folic acid conjugates (Scheme 1B). Both strategies provide reliable control over regioselectivity; however, it was noted that in the former incomplete coupling was observed, necessitating dialysis to remove unreacted pteroic acid, while the latter requires multistep preparation of specialized building blocks.

To address purification challenges, Deberle *et al.* reported a solid phase synthesis strategy for orthogonally protected folate conjugates, enabling simple removal of unreacted materials and byproducts through filtration.^9^ However, a final HPLC purification step is still required. While this approach proved to be effective on a small scale, its reliance on large excesses of reagents limits scalability and environmental sustainability.

Inspired by the work reported by the laboratories of Benvegnu^7^ and Thompson,^6^ we proposed an alternative strategy for regioselective synthesis of γ- folic acid conjugates using an orthogonally protected folic acid building block, as shown in Scheme 1C. In contrast to Thompson’s approach, our design incorporates orthogonal protecting groups at both the γ- and α-carboxylic acid sites: acid-labile *tert*-butyl group and a base-labile methyl ester. This protection strategy enables site-selective deprotection under mild conditions, affording precise control over regioselectivity during subsequent functionalizations. As a result, this building block allows for on-demand installation of γ- and α-conjugates, expanding the scope for generating fit-for-purpose folate regioisomers.

To establish the orthogonal protection strategy, we proposed to assemble orthogonally protected folic acid (**4**) from commercially accessible building blocks, pteroic acid (**2**), and H-Glu­(*t*Bu)-OMe (**3**) (Scheme 2). Dual protection of the γ- and α-carboxyl groups of **3** was considered essential (1) to prevent intramolecular cyclization of **3** between its *N*-α-amino and pendant carboxylic acid functionalities and (2) to suppress its undesired side coupling with the primary amine of pteroic acid. Importantly, this approach also eliminates the need for N-protection of the pteroic acid moiety, which was attempted but proved to be challenging in our hand. Selective γ-deprotection under acidic conditions yields intermediate **5**, exposing the γ-carboxylic acid to site-selective conjugation. Subsequent coupling with functional moieties (**6a–c**) followed by α-deesterification under basic conditions affords the desired γ-conjugated isomers (**8a–c**) with complete regioselective control.

**Scheme 2:**
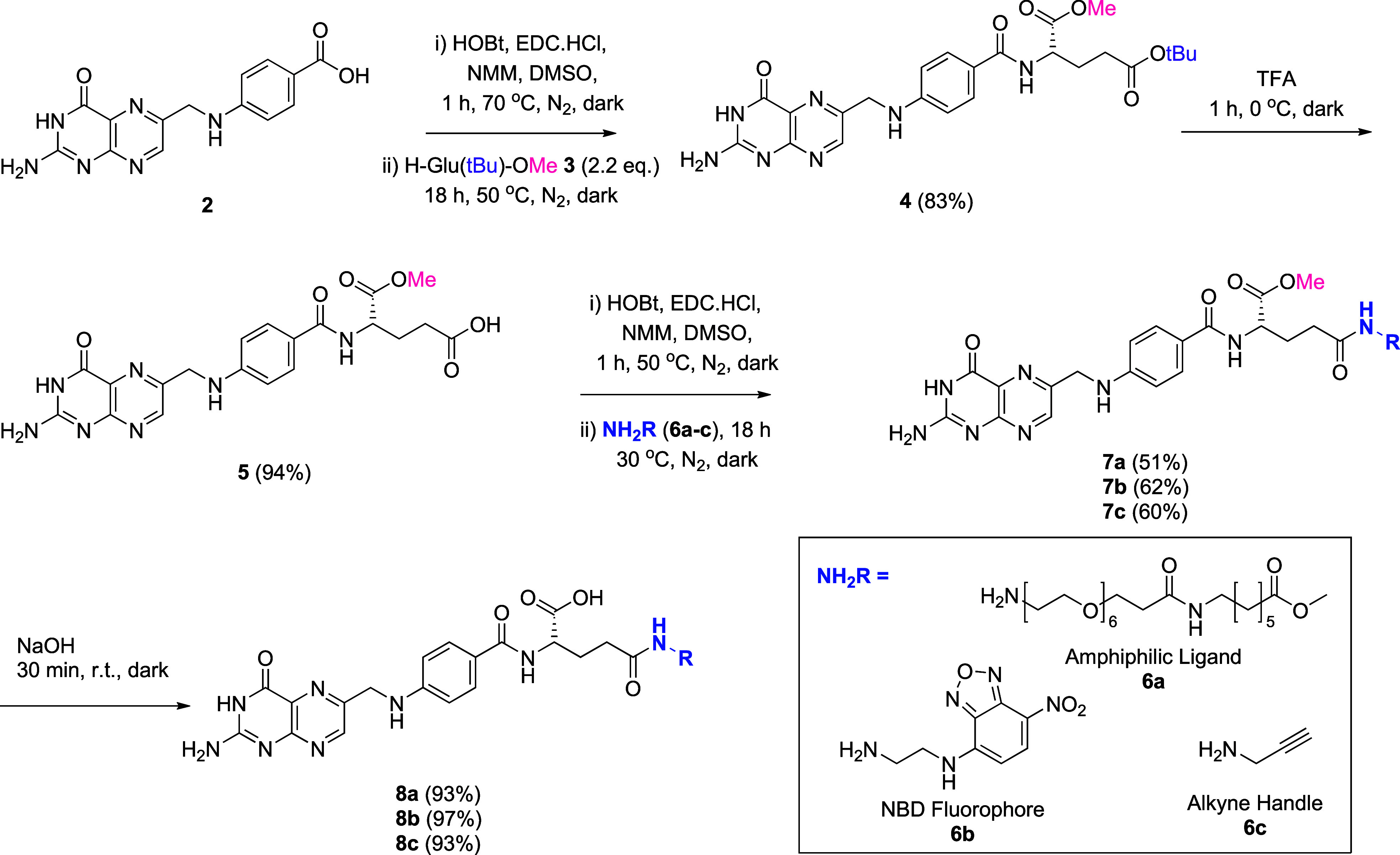
Proposed Orthogonal Route for Regioselective Access to γ-Conjugates of Folic Acid

As control studies, we first investigated the direct coupling of unprotected folic acid **1** with exemplar amphiphilic ligand **6a** to access γ-folate conjugate **8d** (Table 1). The reported conditions^5^ employing carbodiimides such as *N,N′*-dicyclohexylcarbodiimide (DCC), in combination with pyridine or triethylamine as the base, in the presence or absence of the *N*-hydroxysuccinimide (NHS) additive, were investigated. All reactions proceeded sluggishly. Complex regioisomeric mixtures containing γ-, α-, and bis-conjugates, as well as impurities, were generated. These mixtures proved to be challenging to separate, necessitating reverse phase purification and resulting in poor yields (3–8%). Our results are consistent with the observations of Thompson and co-worker*s* ^6^ and further reinforce the necessity of a stepwise approach to achieve regioselective access to γ-folic acid conjugates.

**Table 1:**
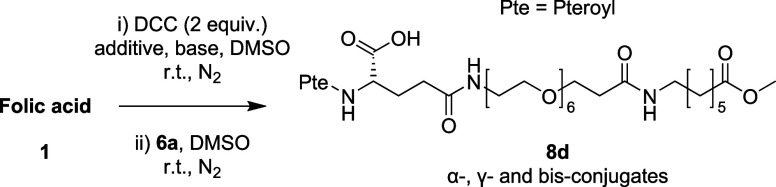
Direct Coupling Using Unmodified Folic Acid **1**

entry	NHS (equiv)	base (equiv)	time (h)	γ:α:bis ratio^a^	yield (%)^b^
1	–	pyridine (50)	18	–	–
2^c^	2	TEA (4)^e^	18	7:2:2	3^d^
3^c^	2	TEA (4)	42	10:3:3	8^d^

a Ratio of γ-, α-, and bis-conjugates determined by LC-MS.

b Isolated yield of γ-conjugates by preparative HPLC.

c Reaction performed in the dark.

d Impurities observed.

e No conversion observed when 2 equiv of TEA used.

As such, we proceeded with the proposed orthogonal protection strategy. Orthogonally protected folic acid **4** was synthesized from **2** and **3** using DCC and 1-hydroxybenzotriazole (HOBt) coupling agents in the presence of 4-methylmorpholine (NMM) as the base (1 h preactivation, Table 2, entry 1), using modified conditions previously reported by the Schirck lab.^10^ The reaction proceeded smoothly with clean conversion (Figure S1), and compound **4** was obtained by precipitation and sequential aqueous/organic washes, which removed most byproducts; however, residual dicyclohexylurea (DCU) persisted. A crude yield of 108% was obtained, albeit with only 32% pure **4**, with DCU being the main impurity.

**Table 2:**
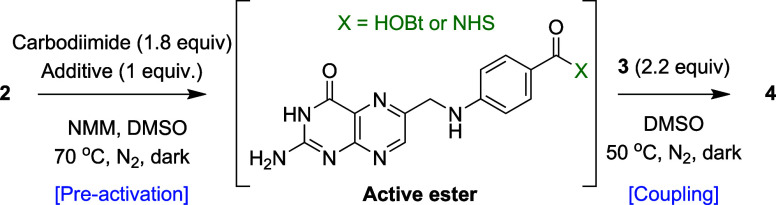
Optimization of Pteroic Acid **2** Activation Time and Coupling Conditions

entry	activation reagent	additive	NMM (equiv)	activation time (h)	yield (%)^a^
1^d^	DCC	HOBt	1.2	1	108^b^
2^d^	EDC	HOBt	1.2	1	78^c^
3^e^	EDC	HOBt	4	1	83^c^
4	EDC	HOBt	4	4	17
5	EDC	HOBt	4	6	11
6	EDC	NHS	4	1	27
7	EDC	NHS	4	4	34
8	EDC	NHS	4	6	36

a Yield determined by ^1^H NMR.

b Crude yield.

c Isolated yield.

d Reaction time of 48 h.

e Reaction completed within 18 h.

To minimize purification requirements, we paired water-insoluble pteroyl compounds with water-soluble coupling reagents to remove coupling byproducts by simple water trituration. Replacing DCC with 1-ethyl-3-(3-(dimethylamino)­propyl)­carbodiimide (EDC) proved to be advantageous, as its water-soluble urea byproduct was readily removed. Although the reaction proceeded smoothly, ∼20% of unreacted pteroic acid remained after 48 h (Table 2, entry 2, and Figure S1).

We next optimized preactivation conditions for generating the active ester of **2** for efficient couplings. Reported procedures for pteroic acid coupling vary in activation reagents, additives, bases, and/or preactivation times. We increased the amount of base and systematically examined two common additives (HOBt vs NHS) and preactivation times (1, 4, and 6 h) for *in situ* generation of pteroic acid active ester (Table 2, entries 3–8, and Figure S2). Increasing the stoichiometry of NMM was advantageous, improving the conversion significantly to 95% within 18 h (entry 3). Protected folic acid (**4**) was isolated in excellent yield (83%) and high purity by ^1^H NMR following repeated water trituration. However, increasing the preactivation time (entries 4 and 5) led to poor conversion to **4** even after a prolonged reaction time, likely due to hydrolysis of the active ester back to **2**. NHS (entries 6–8) was less effective, requiring longer preactivation and still giving reduced yields compared to HOBt.

With orthogonally protected folic acid (**4**) in hand, selective deprotection at the γ-position was achieved under neat TFA at 0 °C, affording **5** in excellent yield. This enabled site-selective γ-conjugation with amines bearing diverse functionalities (**6a–c**) (Scheme 2). We proceeded to access γ-conjugates based on reported folate receptor targeting modalities of interest. Introduction of a PEG-alkylamine linker (**6a**) afforded amphiphilic γ-folic acid conjugate **8a**, suitable for covalent attachment or micelle formation for drug delivery applications.^11^ Incorporation of a nitrobenzodiazole (NBD)-ethylenediamine linker (**6b**) generated fluorescent γ-conjugate **8b**, which is useful for bioimaging studies.^12^ Finally, conjugation with propargylamine (**6c**) furnished **8c**, bearing an alkyne handle amenable for bioorthogonal “click” chemistry.^5c,13^

Compounds **6a** and **6b** were synthesized as outlined in Scheme 3. Ester protection of 7-aminoheptanoic acid,^14^ followed by coupling with a heterofunctional Boc-NH-PEG_6_-CH_2_CH_2_COOH hydrophilic linker and subsequent Boc deprotection, afforded **6a** in excellent yield (82%). Compound **6b** was prepared via nucleophilic aromatic substitution of NBD chloride with *N*-Boc-ethylenediamine, followed by Boc deprotection using reported methods.^15^

**Scheme 3:**
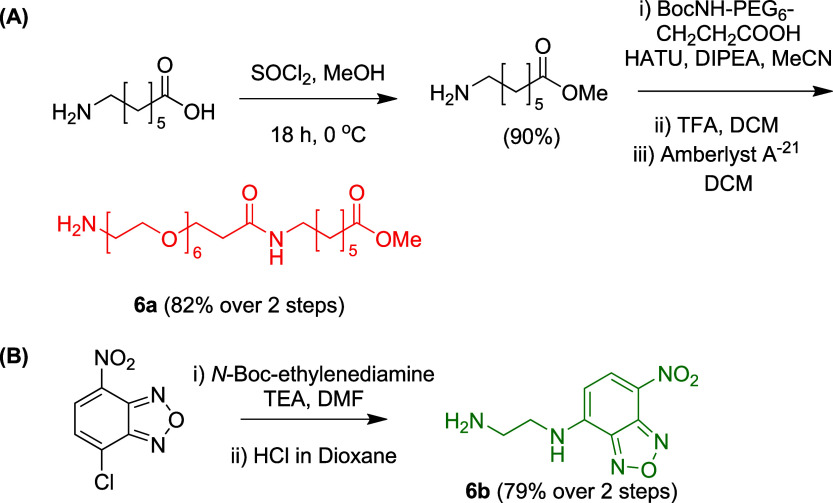
Synthesis of **6a** and **6b**

The coupling of **6a–c** with **5** proceeded smoothly, delivering exclusively γ-conjugated products (**7a–c**, respectively) in good yields (51–62%) (Scheme 2). Notably, the conjugation of **6a** with orthogonally protected folic acid provided absolute regioselectivity and significantly improved yields compared to the use of unprotected folic acid in the control study, which furnished inseparable regioisomeric mixtures (section S5 and Figure S3). Finally, α-ester hydrolysis of **7a–c** with aqueous NaOH, followed by water trituration, afforded spectroscopically pure γ-folic acid conjugates (**8a–c**, respectively) in moderate yields (47–60%) without the need for chromatographic purification (Scheme 4).

**Scheme 4:**
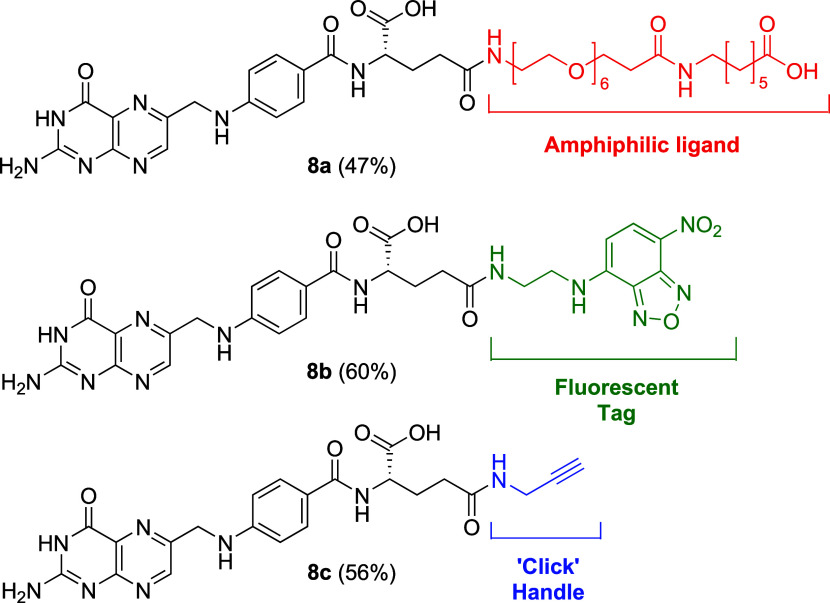
γ-Folic Acid Conjugates Prepared from **5**

Although beyond the scope of this work, we also examined the potential of **4** for orthogonal α-modification of folic acid. Treatment of **4** with stoichiometric NaOH cleanly removed the α-methyl ester while leaving the γ-*tert*-butyl ester intact, giving **9** in excellent yield (Scheme 5). The resulting free α-carboxylic acid provides a handle for further functionalization, enabling access to α-folic acid conjugates. To date, most folate-based modalities have focused on γ-derivatization of folate, partly due to the steric challenge in accessing the α-carboxylic acid for conjugation. However, α-regioisomers have been reported to display enhanced tumor accumulation over their γ-counterparts.^16^ Our approach will provide access to this underexploited class of modalities.

**Scheme 5:**
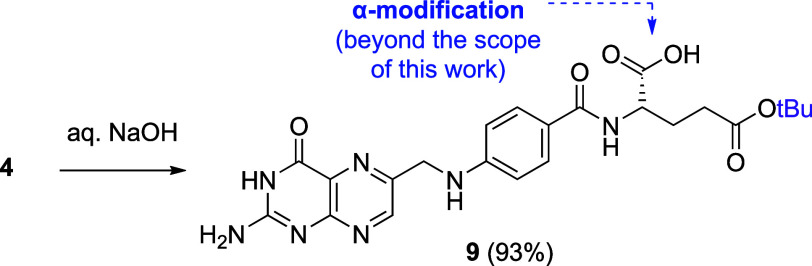
Orthogonal α-Modifications of **4**

In summary, we report an orthogonal strategy that enables regioselective control over both γ- and α-modifications of folic acid. By employing orthogonally γ- and α-protected folic acid as a building block, we achieved site-selective deprotection and then regioselective functionalization, exemplified by the preparation of three distinct classes of γ-conjugates. Orthogonal α-modification was also demonstrated, providing future access to the much less explored α-counterparts. Systematic optimization of coupling conditions of pteroic acid and glutamic acid derivative revealed that EDC/HOBt activation with a 1 h preactivation time affords efficient conversions, while the use of water-soluble reagents enables straightforward purification by aqueous washes and trituration to isolate the spectroscopically pure folic acid species. Collectively, this approach offers regioselective control and synthetic accessibility without the need for reverse phase chromatographic purification to access a variety of functional folate modalities. This method paves the way for on-demand regioselective preparation of folic acid conjugates, thus providing routes to new folate receptor targeting applications in chemical biology in future studies.

## Supplementary Material



## Data Availability

The data underlying this study are available in the published article and its Supporting Information.

## Notes

Experimental procedure and characterization data for compounds and copies of ^1^H and ^13^C NMR spectra and LC-MS data (PDF)
