# Supplementary material for: Orthogonal Regioselective Synthesis of Folic Acid γ‑Conjugates
Source: Org Lett. 2025 Nov 27;27(49):13492–7. doi: 10.1021/acs.orglett.5c04256 (PMC12706834; doi:10.1021/acs.orglett.5c04256)
Supplement: Supplementary file 1 [file ol5c04256_si_001.pdf]

Supporting Information

Orthogonal Regioselective Synthesis of Folic Acid γ-Conjugates.

Ciera F. Connelly and Shiao Y. Chow*

Department of Pure and Applied Chemistry, University of Strathclyde, 295 Cathedral Street, Glasgow G1 1XL, Scotland.

*E-mail: shiao.chow@strath.ac.uk

Table of Contents

S1. General Experimental Details 2

S2. Experimental Procedures and Characterisation Data 2

S3. ^{1}H and ^{13}C NMR Spectra 14

S4. Screening of Optimal Coupling Conditions of 2 and 3 to Access 4. 26

S5. Direct Approach vs Orthogonal Protection Route for Regioselective Conjugation of Exemplar Ligand 6a. 28

S6. HRMS Spectra of Compounds 29

S7. References 40

S1. General Experimental Details

All reagents and starting materials were obtained from commercial sources and used as received. Dry dimethyl sulfoxide and dimethylformamide was obtained from Fisher Scientific. Flash column chromatography was carried out manually using silica gel (Fisher matrix silica 60) with the indicated solvent system. Preparative HPLC was carried out on a Waters XBridge Prep OBD C18 19 x 50 mm, 5 μm column. Merck aluminium-backed plates pre-coated with silica gel 60 (UV254) were used for thin-layer chromatography and visualised by UV light or staining with ninhydrin or an ethanolic solution of phosphomolybdic acid. All NMR spectra were recorded on a Bruker AV500HD (500 MHz) spectrometer at 500 MHz for ^{1}H and 126 MHz for ^{13}C. ^{1}H NMR spectra were referenced to residual DMSO-d$_6$ (δ_H = 2.50 ppm) or $CDCl_3$ (δ_H = 7.27 ppm); ^{13}C NMR spectra were referenced to DMSO-d$_6$ (δ_C = 39.52 ppm) or $CDCl_3$ (δ_C = 77.23 ppm). Data was analysed using MestReNova 10.0 software. Splitting patterns were indicated as singlet (s), broad singlet (br s), doublet (d), doublet of doublet (dd), triplet (t), quartet (q), and multiplet (m). LCMS was carried out on an Agilent Technologies 1220 series LC system with Agilent 6100 series quadrupole mass spectrometer in ESI/APCI mode. The LC was equipped with an Agilent column – Poroshell 120, 4.6 x 7.5 mm, 2.7 μm, C18 with a mobile phase of H_2O + 0.1% formic acid; flow rate:1 mL/min; detection: 214 nm. Results are reported as m/z. HRMS analysis was performed on a ThermoScientific Vanquish UHPLC system connected to a ThermoScientific Exactive Plus Orbi-Trap mass detector. The UHPLC was equipped with a Phenomenex Kinetex 1.7 μm C18 100 Å LC column 30 x 2.1 mm with a mobile phase of MeOH + 0.1% formic acid. Results are reported as m/z.

S2. Experimental Procedures and Characterisation Data

Synthesis of Orthogonally-Protected Folic Acid Building Block

Pteroyl-L-Glu(tBu)-OMe (4) *(5-(tert-butyl) 1-methyl (4-(((2-amino-4-oxo-3,4-dihydropteridin-6-yl)methyl)amino)benzoyl)-L-glutamate)*

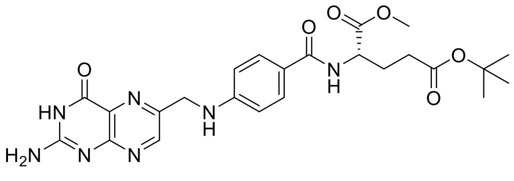

4

In a microwave vial was dissolved pteroic acid (50 mg, 0.16 mmol, 1.0 equiv.), HOBt (22 mg, 0.16 mmol, 1.0 equiv.), EDC.HCl (55 mg, 0.29 mmol, 1.8 equiv.) and *N*-methylmorpholine (70 μL, 0.64 mmol, 4.0 equiv.) in anhydrous DMSO (10 mL), stirring at 70 ºC in heating block, under nitrogen atmosphere. After 1 hour, H-Glu(*t*Bu)-OMe (89 mg, 0.35 mmol, 2.2 equiv.) was dissolved in anhydrous DMSO (2 mL) and added to reaction mixture. The reaction mixture was stirred at 50 ºC

in heating block, under nitrogen atmosphere in darkness. After 18 h, the reaction mixture was poured into ice cold milli-Q H_2O (150 mL) while stirring and then stored in the freezer for 2 hours. The yellow precipitate was then centrifuged at 4400 rpm for 15 minutes and washed with Milli-Q H_2O (2 x 50 mL), followed by a washing with DCM (30 mL). Crude mixture was then dried under *high vac* to obtain **4** as dark yellow/brown powder (0.067 g, 0.13 mmol, 83%) with 97% purity. **^{1}H NMR** (500 MHz, DMSO-d$_6$) δ 8.65 (s, 1H), 8.24 (d, *J* = 7.5 Hz, 1H), 7.65 (d, *J* = 8.4 Hz, 2H), 6.95 (t, *J* = 5.9 Hz, 1H), 6.64 (d, *J* = 8.4 Hz, 2H), 4.49 (br s, 2H), 4.42 – 4.35 (m, 1H), 3.61 (s, 3H), 2.30 (t, *J* = 7.5 Hz, 2H), 2.05 – 1.97 (m, 1H), 1.93 – 1.89 (m, 1H), 1.37 (s, 9H). **^{13}C NMR** (126 MHz, DMSO-d$_6$) δ 172.7, 171.6, 166.4, 153.8, 150.9, 148.6, 148.2, 129.0, 127.9, 121.0, 111.2, 79.8, 51.8, 51.7, 45.9, 31.3, 27.7, 25.9. *Note:* missing quaternary aromatic carbons was observed likely due to overlapping signals and/or insufficient relaxation time. **HMRS** (ESI) *m/z*: $[M + H]^+$ Calcd for $C_{24}H_{30}O_6N_7$ 512.2252; Found 512.2241.

Pteroyl-L-Glu-OMe (5) *((S)-4-(4-(((2-amino-4-oxo-3,4-dihydropteridin-6-yl)methyl)amino)benzamido)-5-methoxy-5-oxopentanoic acid)*

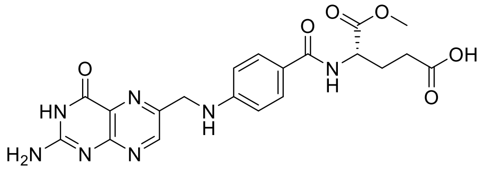

5

4 (55 mg, 0.11 mmol, 1.0 equiv.) was dissolved in *neat* TFA (1 mL) at 0 °C using an ice bath and was allowed to stir for 1 hour. Reaction mixture was reduced to dryness by nitrogen air before the residue was triturated with diethyl ether (2 x 2 mL) and further dried by nitrogen air to obtain **5** as yellow powder which was used in next step without further purification (0.046 g, 0.10 mmol, 94%). **^{1}H NMR** (500 MHz, DMSO-d$_6$) δ 8.68 (s, 1H), 8.26 (d, *J* = 7.3 Hz, 1H), 7.65 (d, *J* = 8.7 Hz, 2H), 7.29 (br s, 1H), 6.64 (d, *J* = 8.7 Hz, 2H), 4.51 (br s, 2H), 4.41 – 4.28 (m, 1H), 3.61 (s, 3H), 2.35 – 2.28 (m, 2H), 2.07 – 2.00 (m, 1H), 1.96 – 1.88 (m, 1H). **^{13}C NMR** (126 MHz, DMSO-d$_6$) δ 173.8, 172.7, 166.4, 153.2, 150.8, 148.3, 129.0, 128.0, 121.0, 111.2, 51.8, 51.7, 45.8, 30.2, 25.8. *Note:* Low S/N ratio and missing quaternary aromatic carbons were observed due to poor solubility of **4** in DMSO-d$_6$. **HMRS** (ESI) *m/z*: $[M + H]^+$ Calcd for $C_{20}H_{22}O_6N_7$ 456.1626; Found 456.1614.

Synthesis of Functional Ligands 6a and 6b

i) Ligand 6a

Methyl 7-Aminoheptanoate

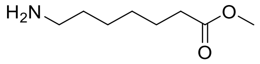

Methyl 7-Aminoheptanoate was synthesized based on method reported in the literature.[1] To a solution of 7-aminoheptanoic acid (515 mg, 3.6 mmol, 1.0 equiv.) in MeOH (10 mL), thionyl chloride (562 µL, 7.8 mmol, 2.2 equiv.) was added dropwise at 0 °C. The resulting mixture was allowed to return to room temperature and left to stir overnight. After 18 hours, reaction mixture was concentrated in vacuo to obtain white solid, washed twice with MeOH and concentrated in vacuo to extract solvent. Residue was then triturated twice using Et_2O to extract biproducts to afford white solid (0.508 g, 3.195 mmol, 90% yield). **^{1}H NMR** (400 MHz, D_2O) δ 3.72 (s, 3H), 3.02 (t, J = 7.4 Hz, 2H), 2.43 (t, J = 7.4 Hz, 2H), 1.73 – 1.61 (m, 4H), 1.44 – 1.35 (m, 4H). **^{13}C NMR** (101 MHz, D_2O) δ 177.2, 51.5, 39.0, 33.2, 26.9, 25.9, 24.6, 23.3. MS (ESI) *m/z*: $[M + H]^+$ Calcd for $C_8H_{18}NO_2$ 160.13; Found 160.20. The NMR spectral data are in agreement with the literature.[2]

NHBoc-(PEG)$_6$-[Alk-6]-COOMe *(methyl 2,2-dimethyl-4,26-dioxo-3,8,11,14,17,20,23-heptaoxa-5,27-diazatetratriacontan-34-oate)*

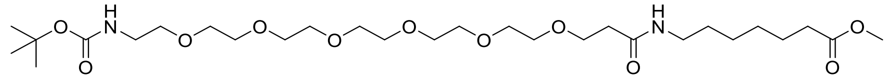

NHBoc-PEG$_6$-CH_2CH_2COOH (500 mg, 1.1 mmol, 1.0 equiv.), DIPEA (422 µL, 2.430 mmol, 2.2 equiv.) and HATU (461 mg, 1.2 mmol, 1.1 equiv.) were dissolved in MeCN (20 mL) and stirred at room temperature. After 15 minutes, methyl 7-aminoheptanoate (259 mg, 1.3 mmol, 1.2 equiv.) in MeCN (10 mL) was added dropwise and reaction mixture was left to stir at room temperature overnight. After 18 hours, the reaction mixture was diluted with EtOAc (25 mL), washed with saturated NaCl (2 x 10 mL), then dried over $MgSO_4$. The reaction mixture was filtered, and the filtrate was concentrated in vacuo. Purification was performed (0 - 10% MeOH in DCM) using column chromatography to afford yellow oil (0.578 g, 0.97 mmol, 88% yield). **^{1}H NMR** (500 MHz, $CDCl_3$) δ 6.61 (br s, 1H), 5.15 (br s, 1H), 3.73 – 3.69 (t, J = 5.6 Hz, 2H), 3.68 – 3.60 (m, 23H), 3.53 (m, 2H), 3.30 (m, 2H), 3.22 (m, 2H), 2.47 (t, J = 5.6 Hz, 2H), 2.32 (m, 2H), 1.63 (m, 2H), 1.50 (m, 2H), 1.44 (s, 9H), 1.37 – 1.33 (m, 4H). **^{13}C NMR** (126 MHz, $CDCl_3$) δ 176.6, 172.0, 70.6, 70.6, 70.6, 70.5, 70.4, 70.3, 67.5, 47.6, 40.5, 39.4, 37.0, 33.9, 29.3, 28.7, 28.6, 26.5, 24.8. **HMRS** (ESI) *m/z*: $[M + H]^+$ Calcd for $C_{28}H_{55}O_{11}N_2$ 595.3800; Found 595.3799.

NH$_2$-(PEG)$_6$-[Alk-6]-COOMe (6a) *(methyl 1-amino-21-oxo-3,6,9,12,15,18-hexaoxa-22-azanonacosan-29-oate)*

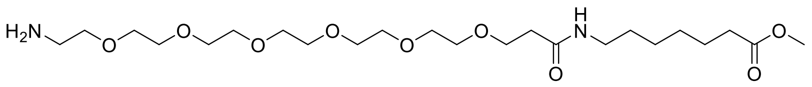

6a

To a solution of NHBoc-(PEG)$_6$-[Alk-$_6$]-COOMe (200 mg, 0.34 mmol, 1.0 equiv.) in DCM (4.5 mL), TFA (500 µL) was added and reaction mixture was allowed to stir for 2 hours at room temperature. Once all starting material was consumed, solvent was removed in vacuo to afford the deprotected product as an oil. The crude oil was redissolved in DCM (5 mL) to which Amberlyst A-21 was added and stirred for 30 minutes to obtain the free base. The reaction mixture was filtered, and the filtrate was concentrated in vacuo to obtain **6a** as yellow oil which was used in next step without further purification (0.154 g, 0.31 mmol, 93% yield). **^{1}H NMR** (500 MHz, $CDCl_3$) δ 7.36 (br s, 1H), 7.11 (br s, 2H), 3.79 (m, 2H), 3.76 (m, 2H), 3.72 – 3.62 (m, 24H), 3.23 (m, 2H), 3.15 (m, 2H), 2.62 (t, J = 5.6 Hz, 2H), 2.33 (t, J = 5.6 Hz, 2H), 1.60 (m, 2H), 1.50 (m, 2H), 1.32 – 1.29 (m, 4H). **HMRS** (ESI) *m/z*: $[M + H]^+$ Calcd for $C_{23}H_{47}O_9N_2$ 495.3276; Found 495.3272.

ii) Ligand 6b

***N*-Boc-NBD-ethylenediamine** *(tert-butyl (2-((7-nitrobenzo[c][1,2,5]oxadiazol-4-yl)amino)ethyl)carbamate)*

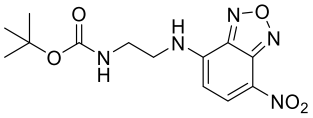

N-Boc-NBD-ethylenediamine was synthesized based on method reported in the literature.[3] To a solution of 4-chloro-7-nitrobenzo[c][1,2,5]oxadiazole (100 mg, 0.50 mmol, 1.0 equiv.) anhydrous DMF (3 mL), tert-butyl (2-aminoethyl)carbamate (89 µL, 0.55 mmol, 1.1 equiv.) was added followed by triethylamine (70 µL, 0.50 mmol, 1.0 equiv.). The reaction mixture was allowed to stir at room temperature, under nitrogen for 4.5 hours. Crude reaction mixture was then added to saturated ammonium chloride solution (20 mL), and extracted with EtOAc (3 x 30 mL). Organic phase was then washed with water (50 mL) and brine (50 mL), and dried over magnesium sulphate, filtered and concentrated in vacuo to yield a dark green/black oil with no further purification required (0.132 g, 0.41 mmol, 82% yield). **^{1}H NMR** (500 MHz, DMSO-d$_6$) δ 9.39 (br s, 1H), 8.53 (d, J = 9.4 Hz, 1H), 7.01 (br s, 1H), 6.44 (d, J = 9.4 Hz, 1H), 3.53 – 3.48 (m, 2H), 3.25 – 3.22 (m, 2H), 1.33 (s, 9H). **^{13}C NMR** (126 MHz, DMSO-d$_6$) δ 155.8, 145.5, 144.5, 144.1, 137.9, 120.8, 99.1, 78.0, 43.3, 35.8, 28.1.

MS (ESI) *m/z*: $[M + H]^+$ Calcd for $C_{13}H_{17}N_5O_5$ 324.1; Found 324.2. The NMR spectral data are in agreement with the literature.[4]

NBD-ethylenediamine (6b) *(N-(7-nitrobenzo[c][1,2,5]oxadiazol-4-yl)ethane-1,2-diamine)*

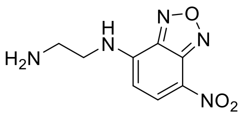

6b

6b was synthesized based on method reported in the literature.[3] *N*-Boc-NBD-ethylenediamine (50 mg, 0.154 mmol, 1.0 equiv.) was dissolved in HCl 2M solution in dioxane (1.2 mL) and stirred overnight under nitrogen atmosphere in the dark at room temperature. After confirming completion of reaction by TLC, the reaction mixture was then dried in vacuo to yield a dark brown oil, to which diethyl ether (2 mL) was added, causing the precipitation of a brown solid, which was collected after centrifugation and washing with diethyl ether then dried under *high vac*. **6b** was used with no further purification required (33 mg, 0.148 mmol, 96% yield). **^{1}H NMR** (500 MHz, DMSO-d_6) δ 9.34 (br s, 1H), 8.58 (d, J = 9.4 Hz, 1H), 8.09 (br s, 2H), 6.53 (d, J = 9.4 Hz, 1H), 3.78 (br s, 2H), 3.16 – 3.12 (m, 2H). MS (ESI) *m/z*: $[M + H]^+$ Calcd for $C_8H_9N_5O_3$ 224.1; Found 224.1. Characterisation in agreement with the literature.[3]

General Procedure 1 (GP1)

In a microwave vial was dissolved Folate-OMe **4** (20 mg, 0.044 mmol, 1.0 equiv.), HOBt (6 mg, 0.044 mmol, 1.0 equiv.), EDC.HCl (15 mg, 0.079 mmol, 1.8 equiv.) and *N*-methylmorpholine (24 µL, 0.18 mmol, 4.0 equiv.) in anhydrous DMSO (5 mL), stirring at 50 °C in heating block, under nitrogen atmosphere. After 1 hour, amine conjugate (**6a - c**) (0.097 mmol, 2.2 equiv.) was dissolved in anhydrous DMSO (1 mL) and added to reaction mixture. The reaction mixture was stirred at 30 °C in heating block, under nitrogen atmosphere in darkness. After 18 hours, the reaction mixture was diluted with milli-Q H_2O (30 mL) and lyophilised to yield crude mixture. Crude solid was then washed by centrifugation at 4400 rpm for 10 minutes with milli-Q H_2O (2 x 10 mL), followed by a washing with DCM (5 mL). Product was then dried under *high vac*.

6a-Fol-OMe (7a) *(dimethyl (S)-2-(4-(((2-amino-4-oxo-3,4-dihydropteridin-6-yl)methyl)amino)benzamido)-5,27-dioxo-9,12,15,18,21,24-hexaoxa-6,28-diazapentatriacontanedioate)*

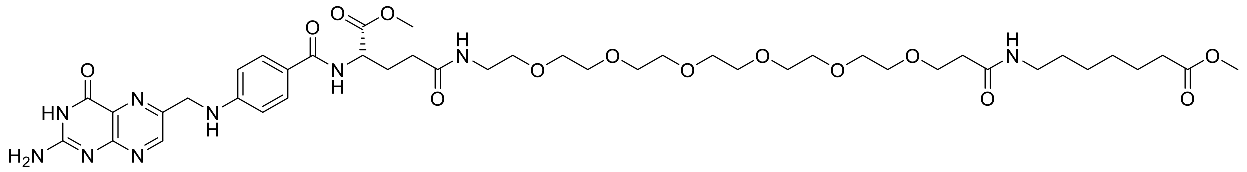

7a

7a was obtained according to GP1 as a yellow/orange solid (0.021 g, 0.023 mmol, 51%). **^{1}H NMR** (500 MHz, DMSO-d$_6$) δ 8.64 (s, 1H), 8.31 (m, 1H), 7.88 (m, 1H), 7.76 (m, 1H), 7.65 (d, *J* = 8.3 Hz, 2H), 6.95 (m, 1H), 6.64 (d, *J* = 8.3 Hz, 2H), 4.48 (br s, 2H), 4.36 – 4.28 (m, 1H), 3.61 – 3.49 (m, 28H), 3.37 – 3.33 (m, 2H; overlapped with water peak), 3.20 – 3.15 (m, 2H), 3.02 – 2.99 (m, 2H), 2.29 – 2.26 (m, 4H), 2.23 – 2.15 (m, 2H), 2.04 – 2.02 (m, 1H), 1.96 – 1.92 (m, 1H), 1.53 – 1.46 (m, 2H), 1.36 (m, 2H), 1.24 – 1.18 (m, 4H). **^{13}C NMR** (126 MHz, DMSO-d$_6$) δ 173.3, 172.8, 171.5, 169.8, 166.3, 154.0, 150.8, 148.7, 129.0, 127.9, 121.0, 111.2, 69.7, 69.7, 69.5, 69.5, 69.1 (overlapped PEG signals), 66.9, 52.3, 51.7, 51.1, 45.9, 38.3, 36.2, 33.2, 31.7, 30.7, 28.9, 28.1, 26.4, 26.0, 24.4. *Note:* missing quaternary aromatic carbons was observed likely due to overlapping signals and/or insufficient relaxation time. **HMRS** (ESI) *m/z*: $[M + H]^+$ Calcd for $C_{43}H_{66}O_{14}N_9$ 932.4724; Found 932.4729.

6b-Fol-OMe (7b) *(methyl N2-(4-(((2-amino-4-oxo-3,4-dihydropteridin-6-yl)methyl)amino)benzoyl)-N5-(2-((7-nitrobenzo[c][1,2,5]oxadiazol-4-yl)amino)ethyl)-L-glutaminate)*

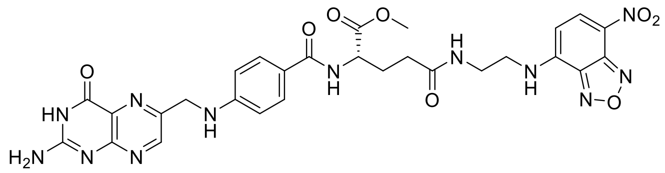

7b

7b was obtained according to GP1 as a dark orange/brown powder (0.018 g, 0.027 mmol, 62%). **^{1}H NMR** (500 MHz, DMSO-d$_6$) δ 8.65 (s, 1H), 8.55 – 8.45 (m, 1H), 8.17 – 8.12 (m, 1H), 7.98 – 7.91 (m, 1H), 7.69 – 7.56 (m, 3H), 6.82 – 6.72 (m, 3H), 6.65 (m, 2H), 6.41 (m, 1H), 4.50 (br s, 2H), 4.46 – 4.34 (m, 1H), 3.72 – 3.52 (m, 5H), 3.51 – 3.33 (m, 2H), 2.24 – 2.15 (m, 2H), 2.13 – 2.02 (m, 1H), 2.00 – 1.89 (m, 1H). **^{13}C NMR** (126 MHz, DMSO-d$_6$) δ 172.4, 171.9, 166.2, 166.1, 153.6, 150.7, 148.3, 144.1, 143.9, 142.5, 137.3, 128.7, 128.5, 127.7, 121.1, 111.1, 111.1, 51.9, 51.4, 45.8, 31.6, 30.1, 26.2, 25.8. *Note:* missing quaternary aromatic carbons was observed likely due to overlapping signals and/or insufficient relaxation time. **HMRS** (ESI) *m/z*: $[M + H]^+$ Calcd for $C_{28}H_{29}O_8N_{12}$ 661.2226; Found 661.2219.

Yne-Fol-OMe (7c) *(methyl N2-(4-(((2-amino-4-oxo-3,4-dihydropteridin-6-yl)methyl)amino)benzoyl)-N5-(prop-2-yn-1-yl)-L-glutaminate)*

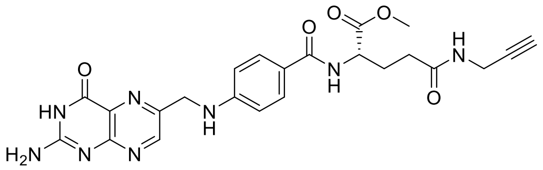

7c

7c was obtained according to GP1 as a yellow powder (0.013 g, 0.026 mmol, 60%). **^{1}H NMR** (500 MHz, DMSO-d$_6$) δ 8.65 (s, 1H), 8.15 (m, 1H), 7.66 (d, *J* = 8.7 Hz, 2H), 6.77 (m, 3H), 6.67 (d, *J* = 8.7 Hz, 2H), 4.50 (br s, 2H), 4.39 – 4.35 (m, 1H), 3.84 (br s, 1H), 3.67 – 3.63 (m, 4H), 3.02 – 2.92 (m, 1H), 2.22 (m, 2H), 2.09 – 2.03 (m, 1H), 1.99 – 1.94 (m, 1H). **^{13}C NMR** (126 MHz, DMSO-d$_6$) δ 172.4, 171.0, 166.2, 153.6, 150.6, 148.3, 128.7, 127.7, 121.2, 111.1, 80.9, 72.4, 52.1, 51.4, 45.8, 31.3, 27.6, 26.2. *Note:* missing quaternary aromatic carbons was observed likely due to overlapping signals and/or insufficient relaxation time. **HMRS** (ESI) *m/z*: $[M + H]^+$ Calcd for $C_{23}H_{25}O_5N_8$ 493.1942; Found 493.1941.

General Procedure 2 (GP2)

Folate-conjugates **7a – c** (0.010 mmol for **7a;** 0.015 mmol for **7b;** 0.020 mmol for **7c**) were dissolved in 1M NaOH aq. solution (0.040 mmol, 4.0 equiv. for **7a**; 0.030 mmol, 2.0 equiv. for **7b;** and 0.040 mmol, 2.0 equiv. for **7c**) and stirred at room temperature for 1 h. After confirming completion of reaction by LCMS, the reaction mixture was precipitated with 1M HCl and the precipitated product was collected by centrifugation. Product was then triturated with milli-Q H_2O (2 x 4 mL) and the precipitated product **8a - c** was collected by centrifugation. Product was lyophilised to remove excess water, with no further purification required.

6a-Fol (8a) *((S)-2-(4-(((2-amino-4-oxo-3,4-dihydropteridin-6-yl)methyl)amino)benzamido)-5,27-dioxo-9,12,15,18,21,24-hexaoxa-6,28-diazapentatriacontanedioic acid)*

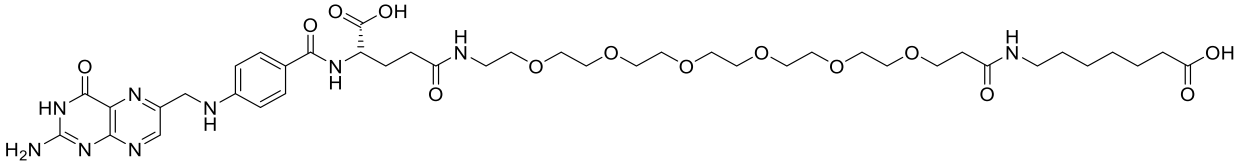

8a

8a was obtained according to GP2 as a dark yellow solid (9.0 mg, 0.010 mmol, 93%) with 96% purity. **^{1}H NMR** (500 MHz, DMSO-d$_6$) δ 8.66 (s, 1H), 8.07 – 7.83 (m, 2H), 7.66 – 7.55 (m, 3H), 6.97 (m, 2H), 6.67 (d, J = 8.7 Hz, 2H), 4.51 (br s, 2H), 4.42 – 4.28 (m, 1H), 3.67 – 3.30 (m, 24H; broadening observed, missing protons likely due to aggregation), 3.23 – 3.20 (m, 2H), 3.05 – 3.01 (m, 2H), 2.29 (m, 2H), 2.19 (m, 2H), 2.09 – 2.02 (m, 1H), 1.98 – 1.90 (m, 1H), 1.51 – 1.48 (m, 2H), 1.46 – 1.37 (m, 2H), 1-33 – 1.25 (m, 4H). **^{13}C NMR** (126 MHz, DMSO-d$_6$) δ 174.5, 174.0, 171.7, 169.8, 166.2, 162.7, 153.8, 151.4, 150.7, 148.6, 128.9, 127.9, 121.4, 111.2, 69.7, 69.5 (overlapping PEG signals), 66.9, 52.3, 45.9, 38.4, 36.2, 33.6, 31.9, 28.9, 28.2, 26.1, 24.4. *Note:* missing quaternary aromatic carbons was observed likely due to overlapping signals and/or insufficient relaxation time. **HMRS** (ESI) *m/z*: [M + H]$^+$ Calcd for $C_{41}H_{62}O_{14}N_9$ 904.4411; Found 904.4400.

6b-Fol (8b) *(N^2-(4-(((2-amino-4-oxo-3,4-dihydropteridin-6-yl)methyl)amino)benzoyl)-N5-(2-((7-nitrobenzo[c][1,2,5]oxadiazol-4-yl)amino)ethyl)-L-glutamine)*

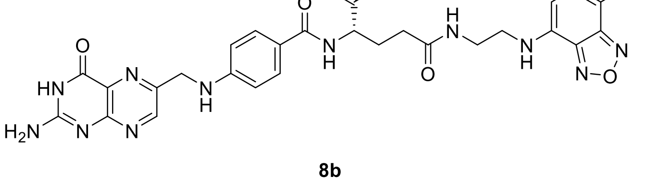

8b

8b was obtained according to GP2 as a dark orange/brown powder (9.5 mg, 0.015 mmol, 97%) with 95% purity. **^{1}H NMR** (500 MHz, DMSO-d$_6$) δ 8.69 (s, 1H), 8.50 (d, J = 8.9 Hz, 1H), 8.06 – 7.91 (m,

2H), 7.69 – 7.62 (m, 2H), 7.51 (br s, 2H), 6.69 – 6.62 (m, 2H), 6.42 (d, J = 8.9 Hz, 1H), 4.54 (br s, 2H), 4.41 – 4.32 (m, 1H), 3.61 – 3.50 (m, 2H), 3.43 – 3.32 (m, 2H), 2.32 – 2.29 (m, 1H), 2.21 – 2.16 (m, 1H), 2.12 – 2.02 (m, 1H), 1.98 – 1.91 (m, 1H). **^{13}C NMR** (126 MHz, DMSO-d$_6$) δ 173.9, 173.7, 166.3, 159.6, 152.9, 150.6, 148.0, 145.4, 144.7, 137.9, 129.0, 128.7, 128.0, 121.4, 111.2, 52.0, 45.8, 31.9, 30.4, 26.4, 26.0. *Note:* missing quaternary aromatic carbons was observed likely due to overlapping signals and/or insufficient relaxation time. **HMRS** (ESI) *m/z*: [M + H]$^+$ Calcd for $C_{27}H_{27}O_8N_{12}$ 647.2069; Found 647.2064.

Yne-Fol (8c) (*N^2-(4-(((2-amino-4-oxo-3,4-dihydropteridin-6-yl)methyl)amino)benzoyl)-N5-(prop-2-yn-1-yl)-L-glutamine)*

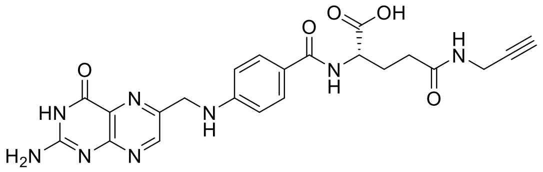

8c

8c was obtained according to GP2 as a yellow powder (9.0 mg, 0.019 mmol, 93%) with 96% purity. **^{1}H NMR** (500 MHz, DMSO-d$_6$) δ 8.68 (s, 1H), 8.18 – 7.88 (m, 2H), 7.66 (d, J = 8.7 Hz, 2H), 7.23 (m, 2H), 6.67 (d, J = 8.7 Hz, 2H), 4.53 (br s, 2H), 4.40 – 4.30 (m, 1H), 4.00 – 3.78 (m, 2H), 3.03 – 2.97 (m, 1H), 2.32 – 2.28 (m, 1H), 2.26 – 2.19 (m, 1H), 2.11 – 2.00 (m, 1H), 1.99 – 1.89 (m, 1H). **^{13}C NMR** (126 MHz, DMSO-d$_6$) δ 173.8, 171.3, 166.3, 160.1, 153.2, 150.7, 148.2, 129.0, 128.0, 121.4, 111.2, 81.2, 72.9, 52.1, 45.8, 31.7, 30.4, 27.8, 26.4. *Note:* missing quaternary aromatic carbons was observed likely due to overlapping signals and/or insufficient relaxation time. **HMRS** (ESI) *m/z*: [M + H]$^+$ Calcd for $C_{22}H_{23}O_5N_8$ 479.1786; Found 479.1780.

Pteroyl-L-Glu(*t*Bu) (9) *(2-(4-(((2-amino-4-oxo-3,4-dihydropteridin-6-yl)methyl)amino)benzamido)-5-(tert-butoxy)-5-oxopentanoic acid)*

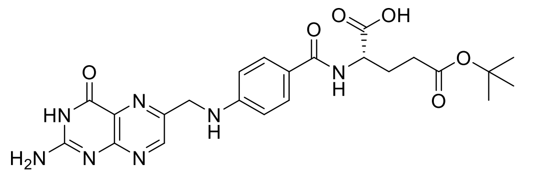

9

4 (10 mg, 0.0196 mmol, 1 equiv.) was dissolved in 1M NaOH aq. (40 μL, 0.039 mmol, 2 equiv.) and milli-Q H_2O (960 μL) and was allowed to stir for 30 minutes at room temperature. After confirming completion of the reaction by LCMS, the reaction mixture was treated with aq. 1M HCl solution and then triturated with milli-Q H_2O (2 x 2 mL) and the precipitated product was collected by

centrifugation. Product was dried under *high vac*, and **9** was obtained with no further purification required as a dark yellow powder (9 mg, 0.018 mmol, 93%) with 96% purity. **^{1}H NMR** (500 MHz, DMSO-d$_6$) δ 8.64 (s, 1H), 8.13-8.08 (m, 1H), 7.68 – 7.62 (m, 2H), 7.14 – 7.09 (m, 1H), 6.97 – 6.87 (m, 2H), 6.64 (d, J = 8.5 Hz, 2H), 4.48 (br s, 2H), 4.35 – 4.28 (m, 1H), 2.32 – 2.24 (m, 2H), 2.08 – 1.97 (m, 1H), 1.89 – 1.85 (m, 1H), 1.37 (s, 9H). **^{13}C NMR** (126 MHz, DMSO-d$_6$) δ 173.7, 171.6, 167.4, 166.3, 153.7, 151.9, 150.8, 148.5, 131.1, 128.0, 121.3, 111.3, 79.7, 51.6, 45.8, 31.6, 27.7, 26.1. **HMRS** (ESI) *m/z*: $[M + H]^+$ Calcd for $C_{23}H_{28}O_6N_7$ 498.2096; Found 498.2086.

<u>Control studies: Direct coupling of unprotected folic acid to access 8d</u>

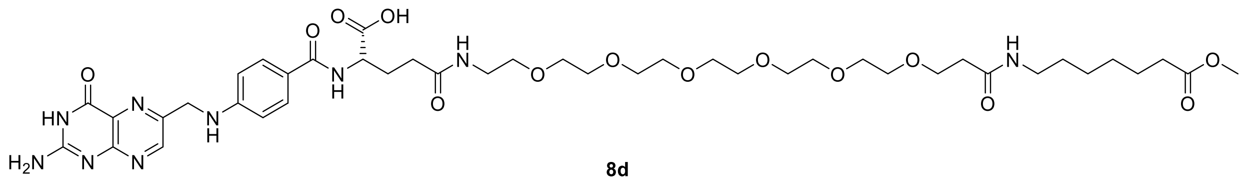

8d

Direct coupling of unmodified folic acid was carried out based on reported methods.[5,6]

Entry 1[5]: Folic acid (**1**) (40 mg, 0.091 mmol, 1.0 equiv.) was dissolved in anhydrous DMSO. To which DCC (37 mg, 0.18 mmol, 2.0 equiv.) and pyridine (490 µL, 4.5 mmol, 50 equiv.) were added and the mixture was stirred for 30 minutes at room temperature. **6a** (54 mg, 0.11 mmol, 1.2 equiv.) in anhydrous DMSO was then added to activated folic acid and reaction mixture was stirred under nitrogen in darkness at room temperature for 18 h.

Entry 2[6]: Folic acid (**1**) (50 mg, 0.11 mmol, 1.0 equiv.) was dissolved in anhydrous DMSO. DCC (47 mg, 0.23 mmol, 2.0 equiv.) and NHS (26 mg, 0.23 mmol, 2.0 equiv.) were added successively. Reaction mixture was stirred for 18 h in darkness, under nitrogen at room temperature, after which the urea precipitate was filtered off. **6a** (66 mg, 0.13 mmol, 1.2 equiv.) and triethylamine (62µL, 0.45 mmol, 4 equiv.) in anhydrous DMSO was added to activated folic acid. The reaction mixture was stirred under nitrogen in darkness at room temperature for 18 h. *Note:* Reaction was also attempted using 2 equiv of TEA albeit with no conversion observed.

Entry 3[6]: Folic acid (**1**) (25 mg, 0.057 mmol, 1.0 equiv.) was dissolved in anhydrous DMSO. DCC (23 mg, 0.11 mmol, 2.0 equiv.) and NHS (13 mg, 0.11 mmol, 2.0 equiv.) were added successively. Reaction mixture was stirred for 18 h in darkness, under nitrogen at room temperature, after which the urea precipitate was filtered off. **6a** (33 mg, 0.067 mmol, 1.2 equiv.) and triethylamine (31 µL, 0.22 mmol, 4 equiv.) were dissolved in anhydrous DMSO and added to activated folic acid solution. The reaction mixture was stirred under nitrogen in darkness at room temperature for 42 h.

Work-up: Reaction mixture was added to a solution of cold 20% acetone in Et_2O and stirred for 10 minutes on ice. The yellow precipitate was carefully centrifuged and washed (2 x acetone; 1 x Et_2O), then dried under *high vac* to afford yellow solid. Complex regioisomeric mixtures were obtained: γ-Conjugate was the favoured product however significant amounts of alpha and bis conjugates were also formed (**Figure S1**).

γ-Conjugate **8d** was isolated by RP-HPLC (injection of sample dissolved in DMSO/H_2O, 8 ml/min, 0 to 4.5 min 95% water, linear gradient from 4.5 min at 95% water to 16 min at 95% acetonitrile, Waters XBridge Prep OBD C18 19 x 50 mm, 5 μm Column) and lyophilised to give yellow solid. **Entry 2**: 3.2 mg, 0.0034 mmol, 3% yield with 71% purity; **Entry 3**: 4.3 mg, 0.0047 mmol, 8% yield with 76% purity. *Note:* Impurities presence was noted despite chromatographic purification. ^{1}H NMR spectra were obtained but not ^{13}C NMR spectra due yield constraints. **^{1}H NMR** (500 MHz, DMSO-d_6) δ 8.67 (s, 1H), 7.77 – 7.64 (m, 2H), 6.93 (*br* s, 2H), 6.69 – 6.63 (m, 2H), 4.49 (br s, 2H), 4.40 – 4.24 (m, 1H), 3.59 – 3.47 (m, 30H; overlapping PEG chain, methyl-CH_3 and water signals), 3.21 – 3.12 (m, 2H), 3.02 – 2.98 (m, 2H), 2.29 – 2.26 (m, 4H), 1.51 – 1.48 (m, 2H), 1.37 – 1.34 (m, 2H), 1.24 - 1.23 (m, 4H). MS (ESI) *m/z*: $[M + H]^+$ Calcd for $C_{42}H_{64}N_9O_{14}$ 918.5; Found 918.4.

Scaled-up synthesis of 8c

Step 1: In a round bottom flask was dissolved pteroic acid (350 mg, 1.1 mmol, 1.0 equiv.), HOBt (152 mg, 1.1 mmol, 1.0 equiv.), EDC.HCl (387 mg, 2.0 mmol, 1.8 equiv.) and *N*-methylmorpholine (495 µL, 4.5 mmol, 4.0 equiv.) in anhydrous DMSO (70 mL), stirring at 70°C in a heating mantle, under nitrogen atmosphere. After 1 hour, H-Glu(tBu)-OMe (625 mg, 2.5 mmol, 2.2 equiv.) in anhydrous DMSO (15 mL) was added and stirred at 50°C in heating block, under nitrogen atmosphere in darkness. After 18 h, the reaction mixture was poured into ice cold milli-Q H_2O (500 mL) while stirring and then stored in the freezer for 2 hours. The yellow precipitate was centrifuged at 4400 rpm for 30 minutes and washed with Milli-Q H_2O (2 x 100 mL) followed by DCM (50 mL). Product was dried under *high vac* to obtain **4** as a dark yellow solid (405 mg, 0.79 mmol, 71%).

Step 2: 4 (400 mg, 0.78 mmol, 1.0 equiv.) was dissolved in TFA (5 mL) at 0 ºC using an ice bath and stirred for 1 hour. TFA was removed by nitrogen, and the crude product was triturated with diethyl ether (2 x 5 mL) then dried by nitrogen to give **5** as a dark yellow powder (332 mg, 0.73 mmol, 93%).

Step 3: In a round bottom flask was dissolved **5** (330 mg, 0.73 mmol, 1.0 equiv.), HOBt (98 mg, 0.73 mmol, 1.0 equiv.), EDC.HCl (250 mg, 1.3 mmol, 1.8 equiv.) and *N*-methylmorpholine (405 µL, 2.9 mmol, 4.0 equiv.) in anhydrous DMSO (40 mL), stirring at 50°C in a heating mantle, under nitrogen atmosphere. After 1 hour, **6c** (100 µL, 1.6 mmol, 2.2 equiv.) in anhydrous DMSO (5 mL) was added to reaction mixture and stirred at 30°C in a heating mantle, under nitrogen atmosphere in darkness. After 18 h, the reaction mixture was poured into ice cold milli-Q H_2O (500 mL) while stirring and then stored in the freezer for 2 hours. The brown precipitate was then centrifuged at 10,000 rpm for 30 minutes and washed with Milli-Q H_2O (2 x 100 mL) followed by DCM (50 mL). Product was then dried under *high vac* to obtain **7c** as a brown powder (202 mg, 0.41 mmol, 57%).

Step 4: 7c (200 mg, 0.41 mmol, 1 equiv.) was treated with 1M NaOH aq. Solution (800 µL, 0.81 mmol, 2 equiv.) in milli-Q H_2O (39.2 mL) and stirred at room temperature for 1 h. The reaction mixture was precipitated with 1M HCl and the product was collected by centrifugation. Product was then washed with milli-Q H_2O (2 x 15 mL) and lyophilised to remove excess water, with no further purification required. Isolated product (178 mg, 0.37 mmol, 92%) was obtained after lyophilisation.

Table S1. Compound **8c** scaling studies

Step	ID	Small-scale		Scaled up	
		Scale (mmol)	Experimental Yield	Scale (mmol)	Experimental Yield
1	4	0.16	67 mg, 0.13 mmol, 83%	1.1	405 mg, 0.79 mmol, 71%
2	5	0.11	46 mg, 0.10 mmol, 94%	0.78	332 mg, 0.73 mmol, 93%
3	7c	0.044	13 mg, 0.026 mmol, 60%	0.73	202 mg, 0.41 mmol, 57%
4	8c	0.020	9 mg, 0.019 mmol, 93%	0.41	178 mg, 0.37 mmol, 92%
		Overall yield:	**44%**	Overall yield:	**35%**

S3. ^{1}H and ^{13}C NMR Spectra

Pteroyl-L-Glu(tBu)-OMe (Compound **4**) – ^{1}H NMR (500 MHz), ^{13}C NMR (126 MHz) in DMSO-d_6

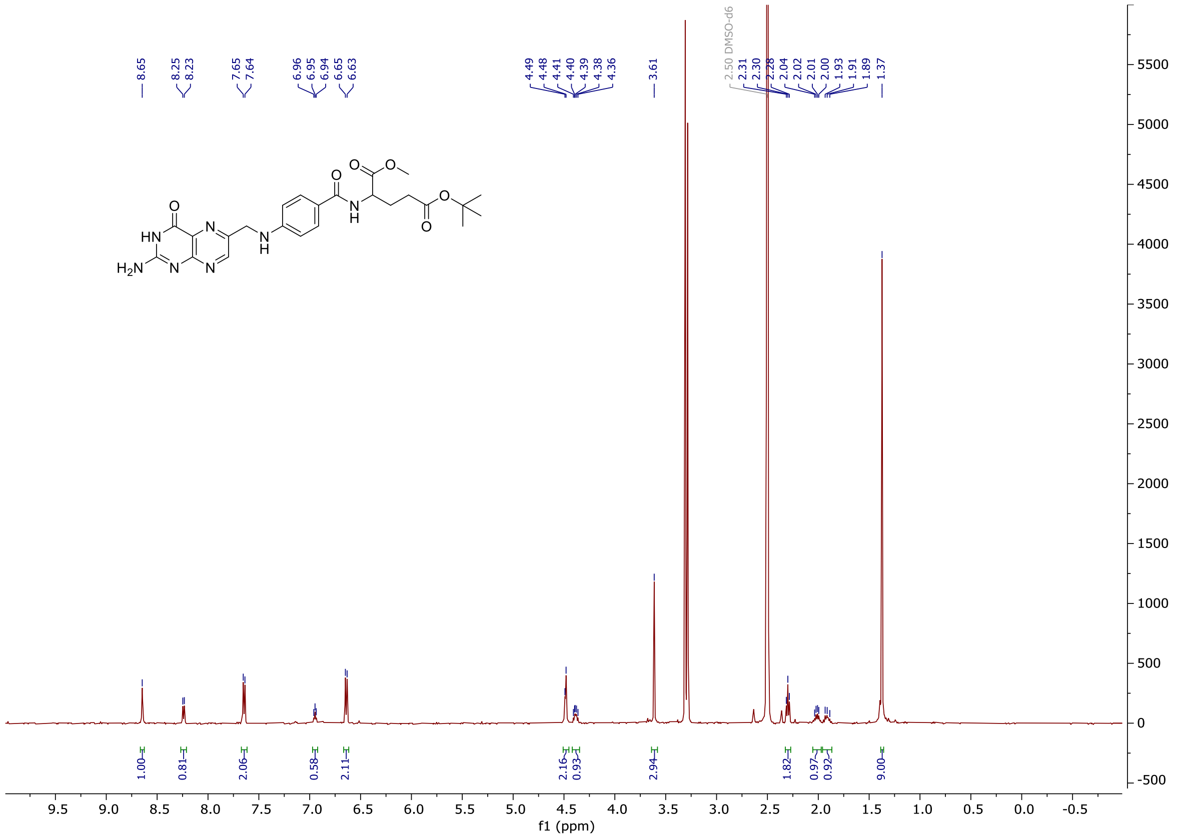

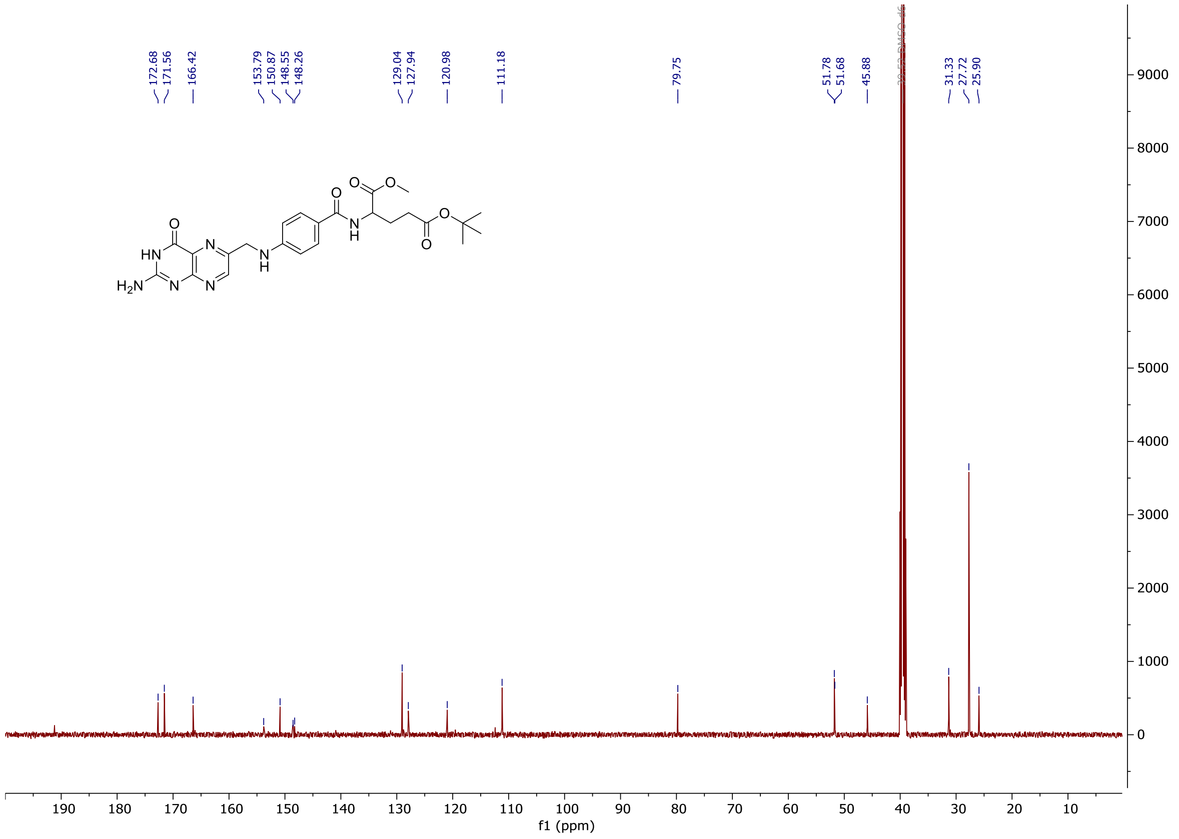

Pteroyl-L-Glu-OMe (Compound **5**) – ^{1}H NMR (500 MHz), ^{13}C NMR (126 MHz) in DMSO-d$_6$

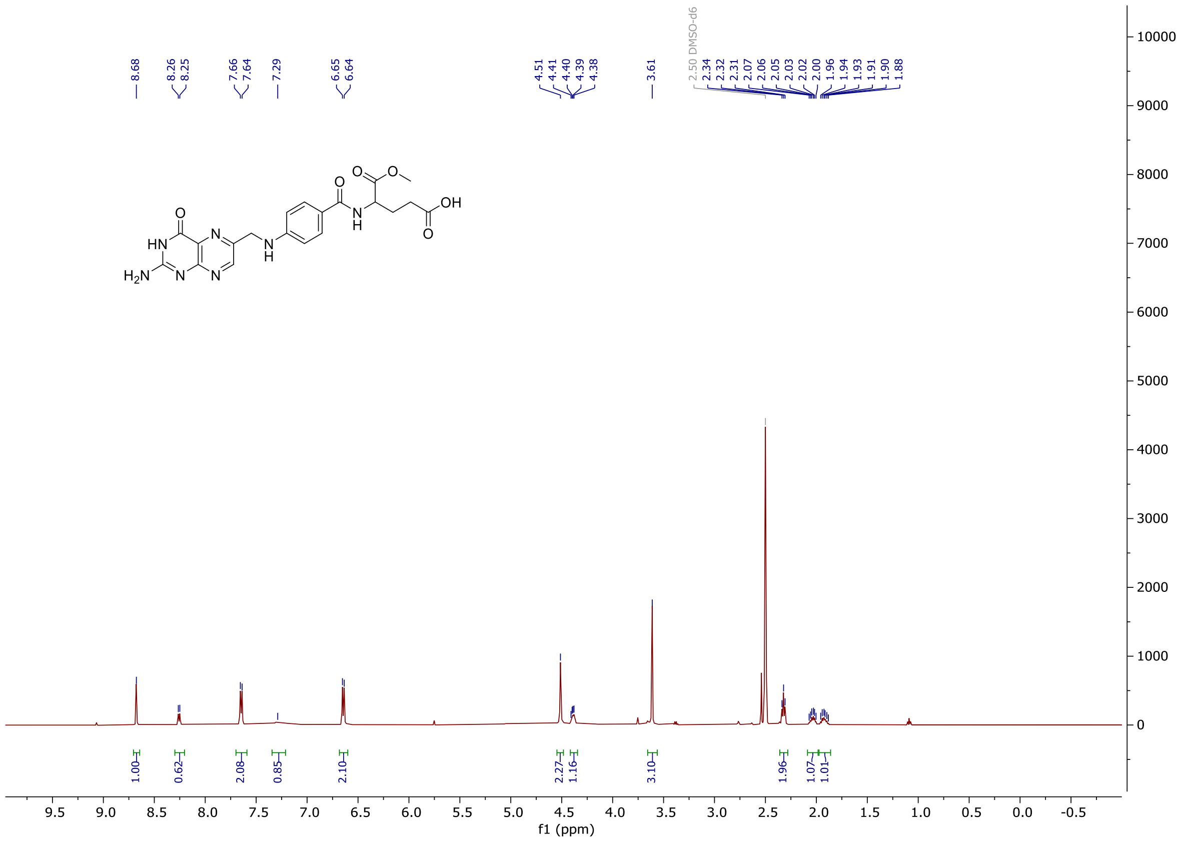

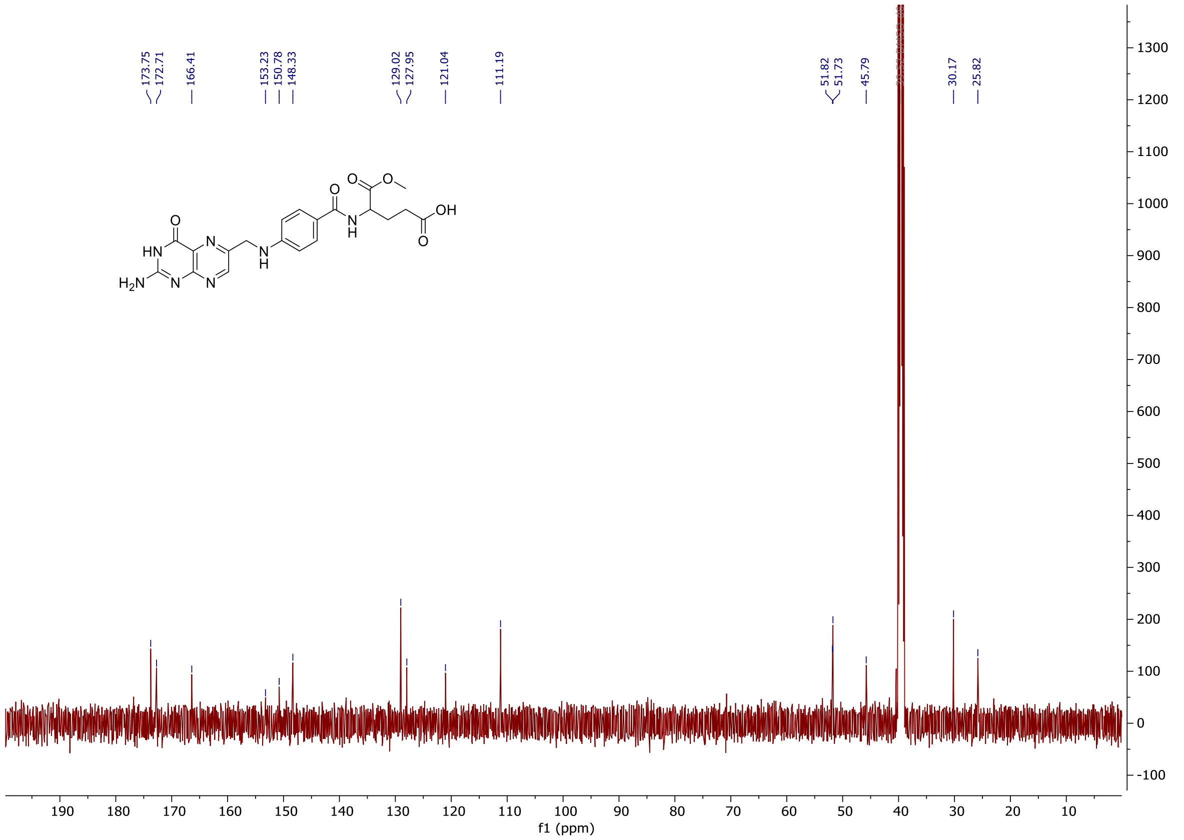

NHBoc-(PEG)$_6$-[Alk-6]-COOMe – ^{1}H NMR (500 MHz), ^{13}C NMR (126 MHz) in $CDCl_3$

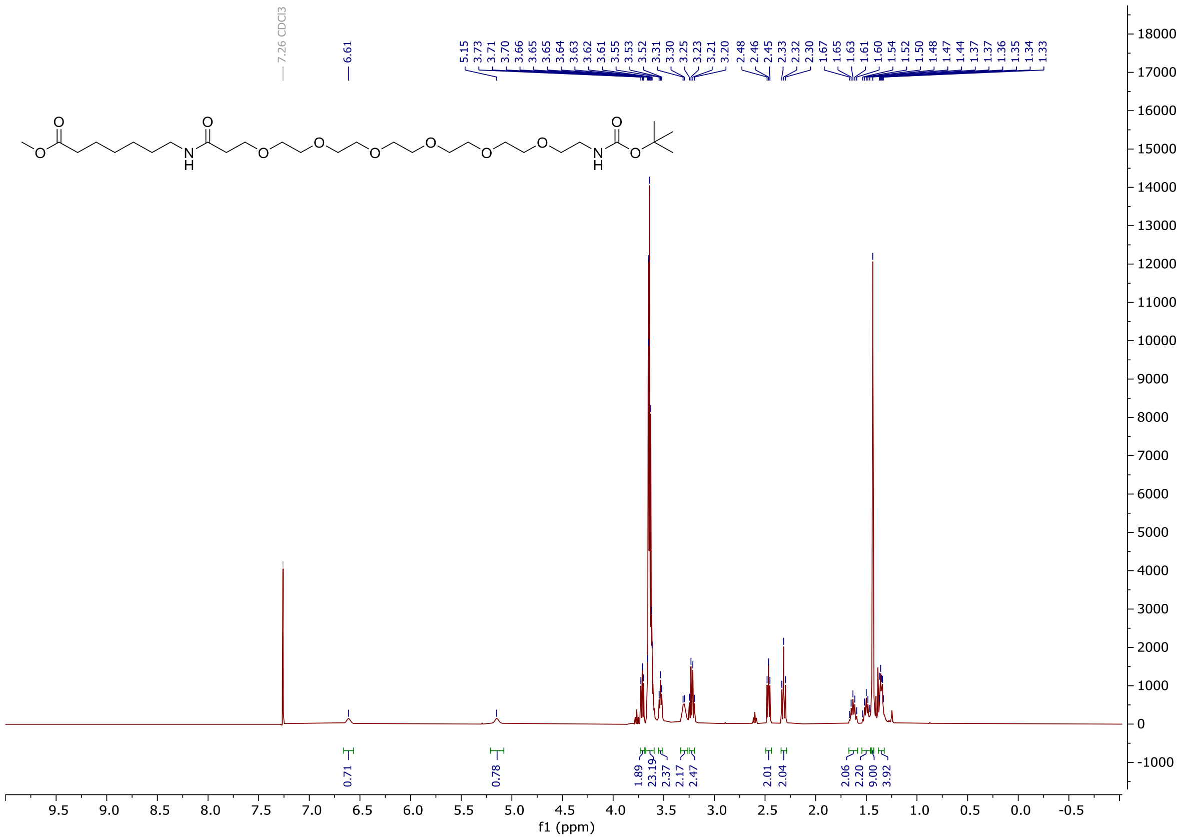

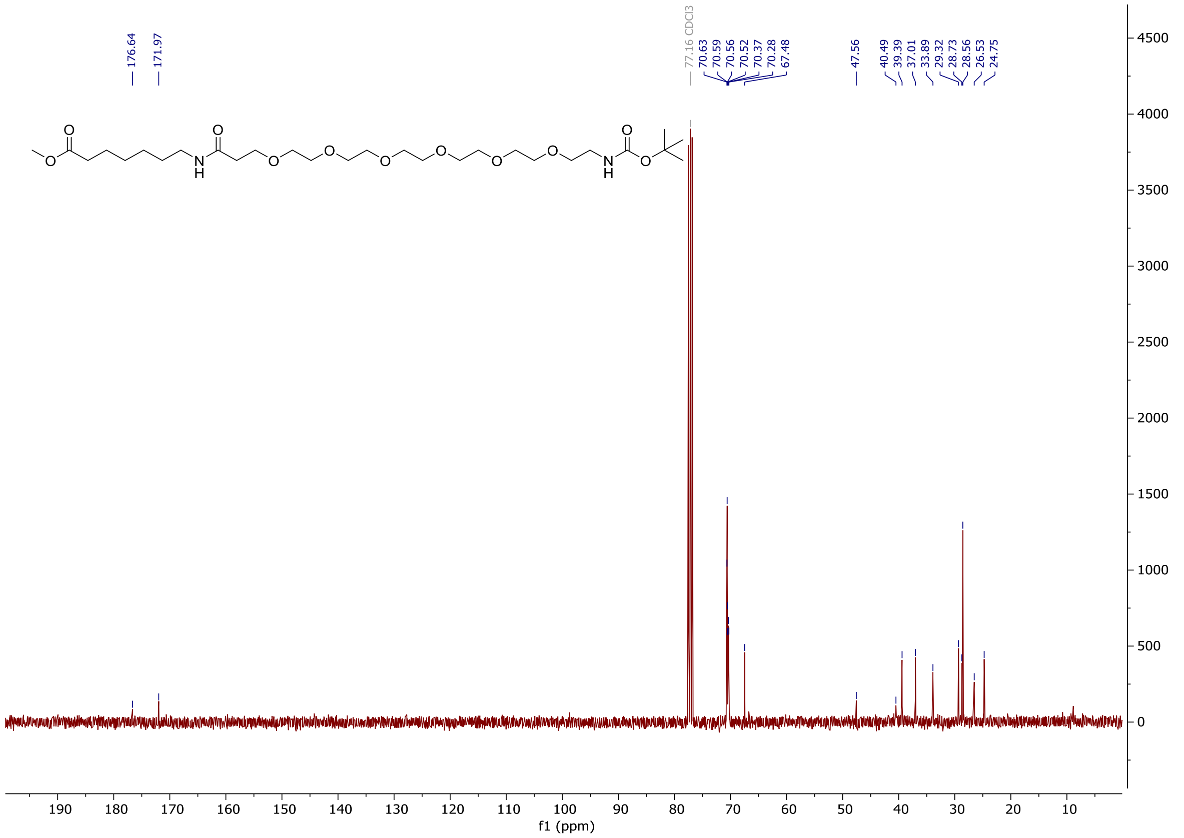

N-Boc-NBD-ethylenediamine – ^{1}H NMR (500 MHz), ^{13}C NMR (126 MHz) in DMSO-d$_6$

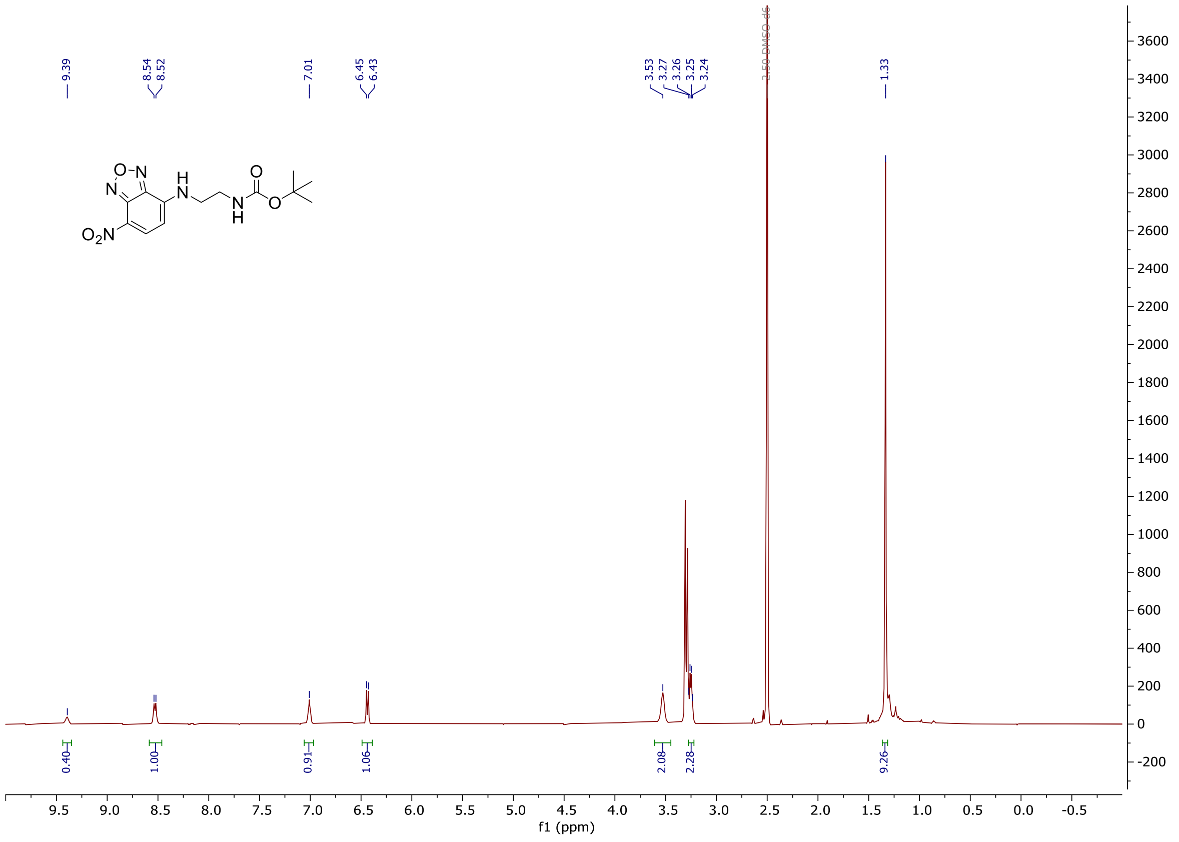

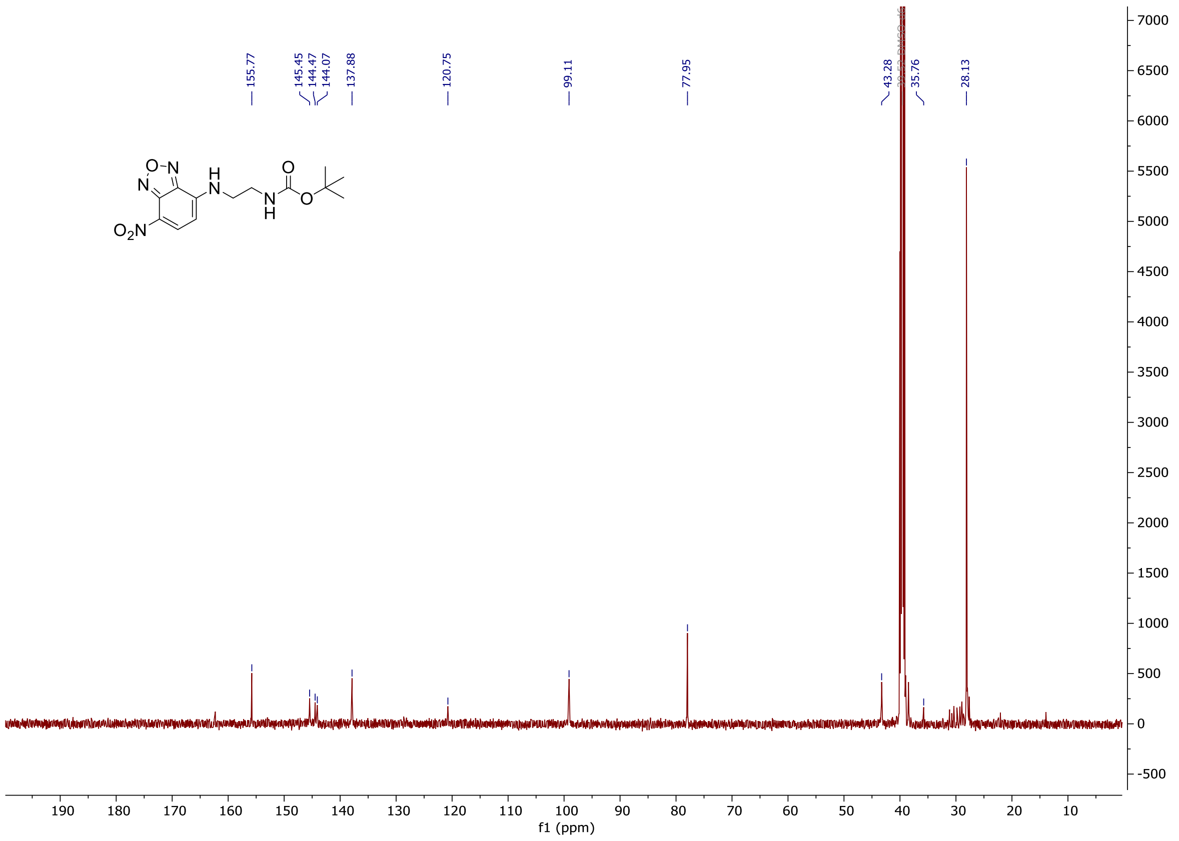

6a-Fol-OMe (Compound **7a**) – ^{1}H NMR (500 MHz), ^{13}C NMR (126 MHz) in DMSO-d_6

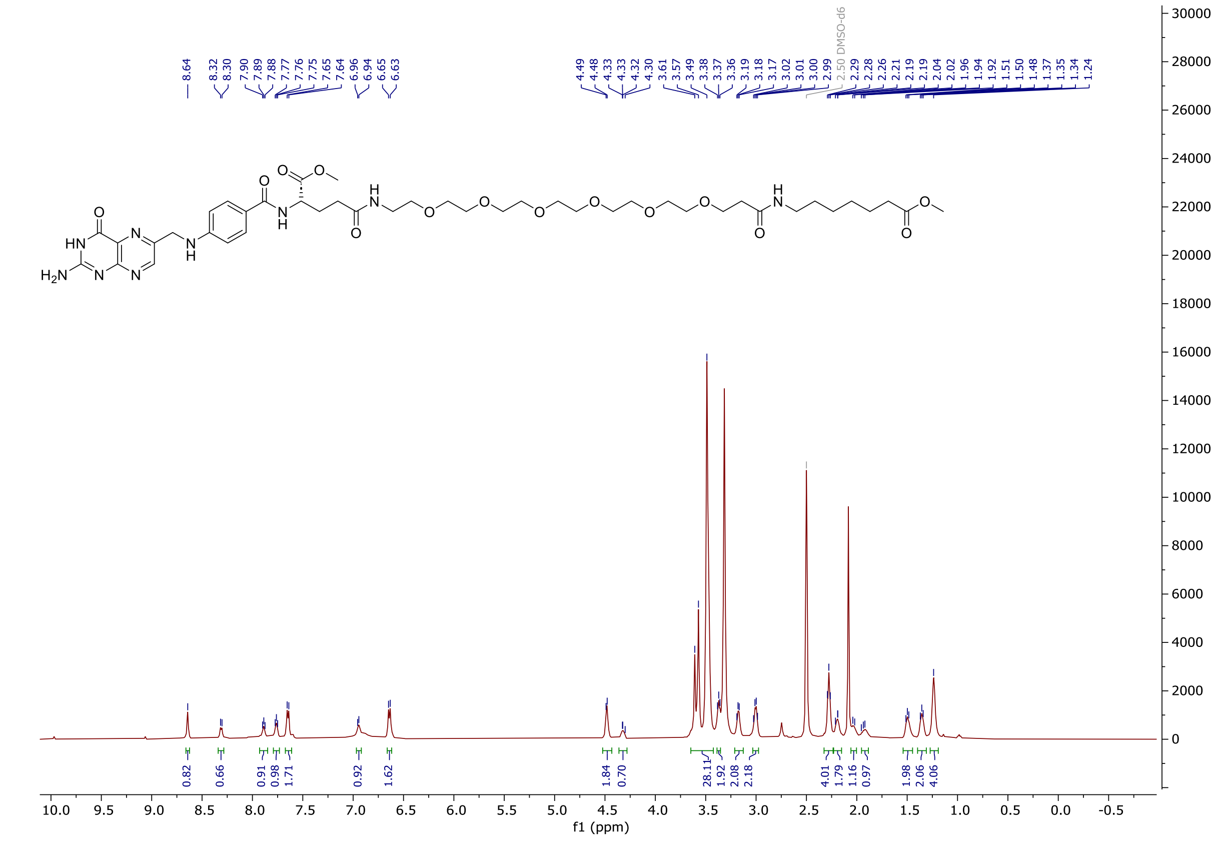

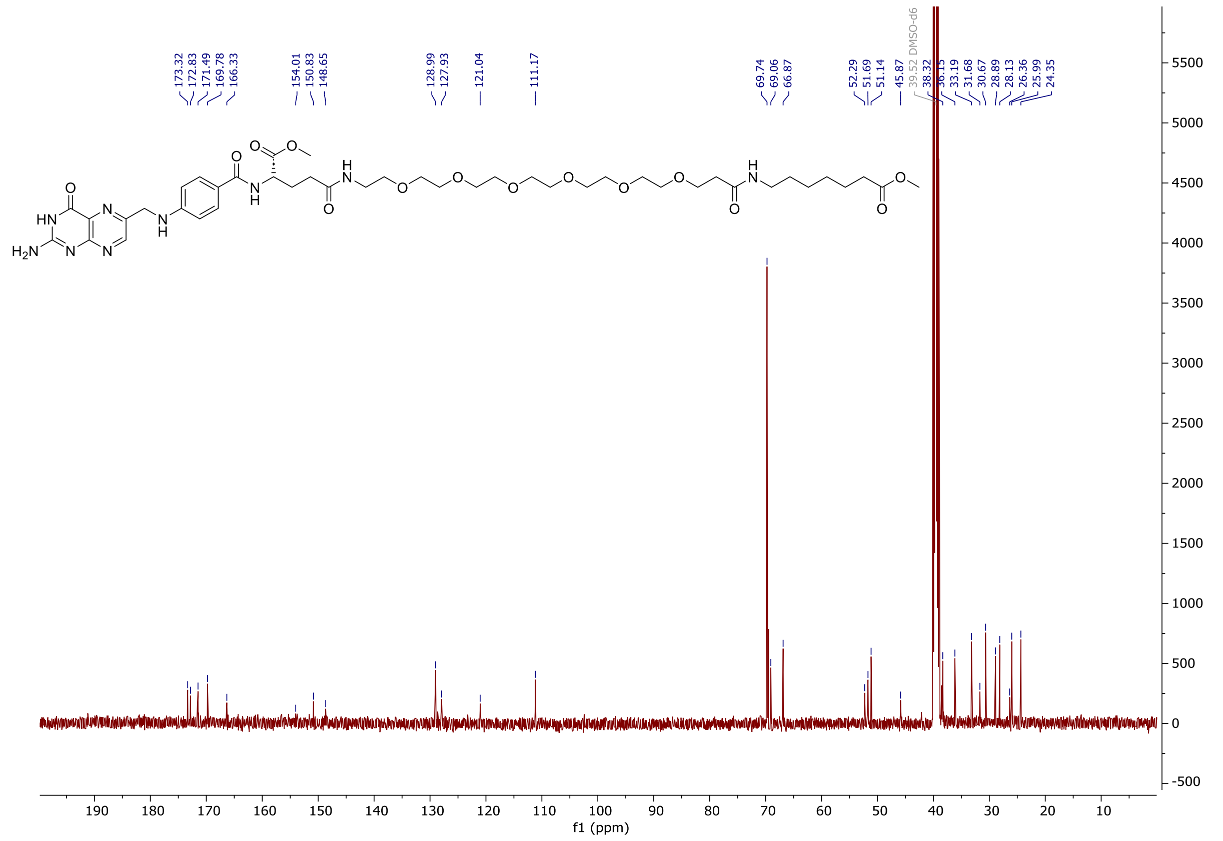

6b-Fol-OMe (Compound **7b**) – ^{1}H NMR (500 MHz), ^{13}C NMR (126 MHz) in DMSO-d_6

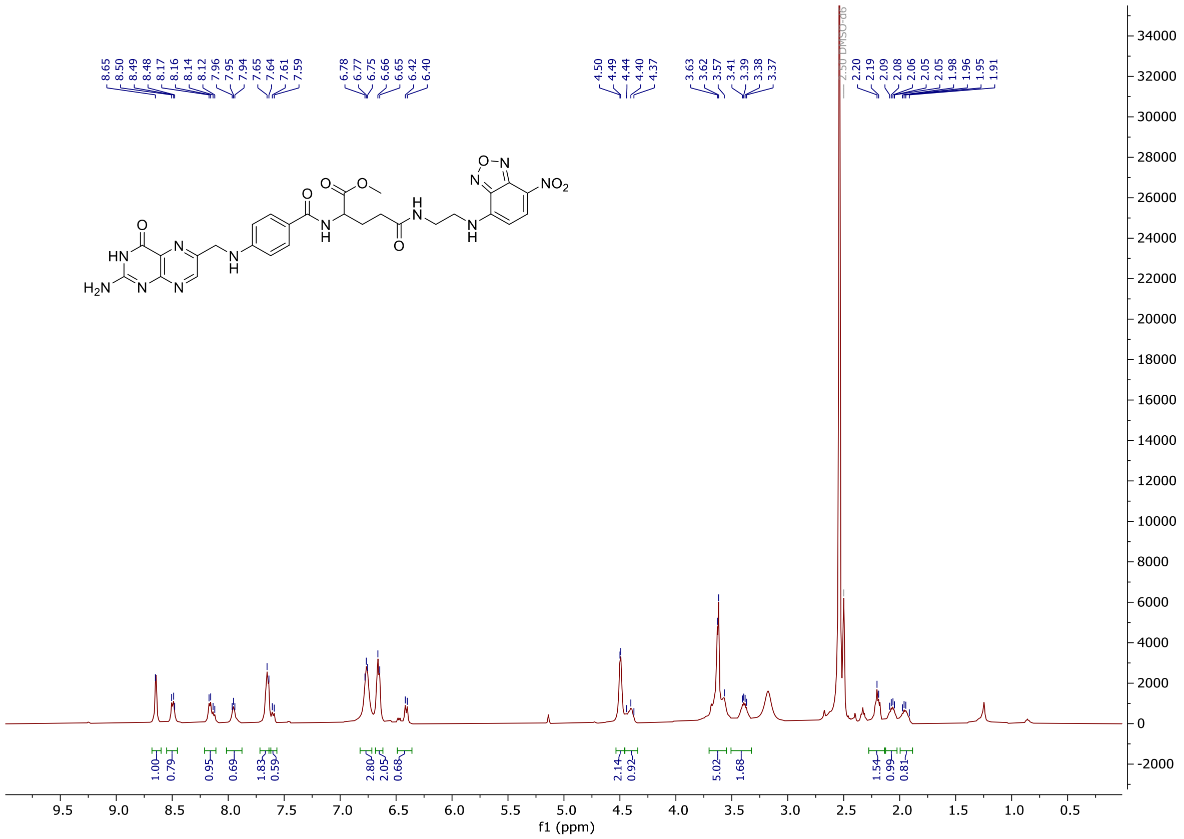

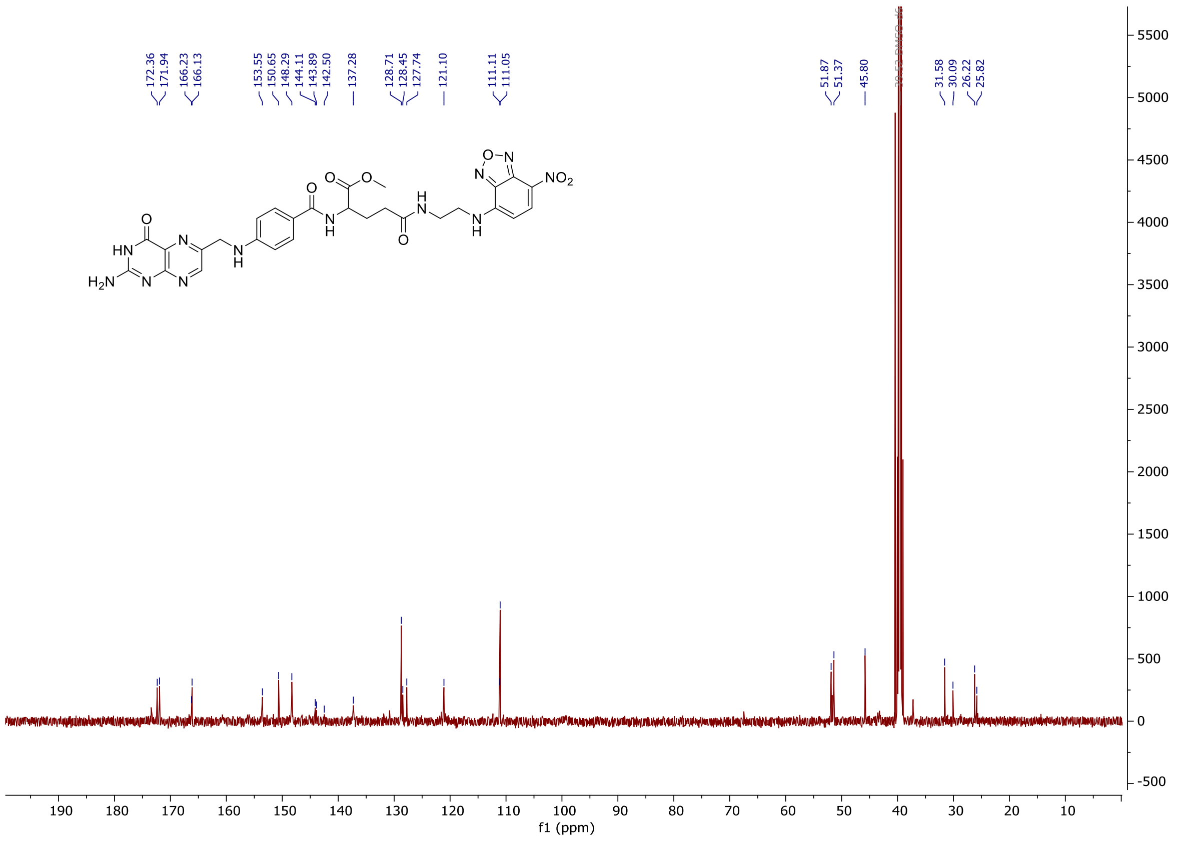

Yne-Fol-OMe (Compound **7c**) – ^{1}H NMR (500 MHz), ^{13}C NMR (126 MHz) in DMSO-d_6

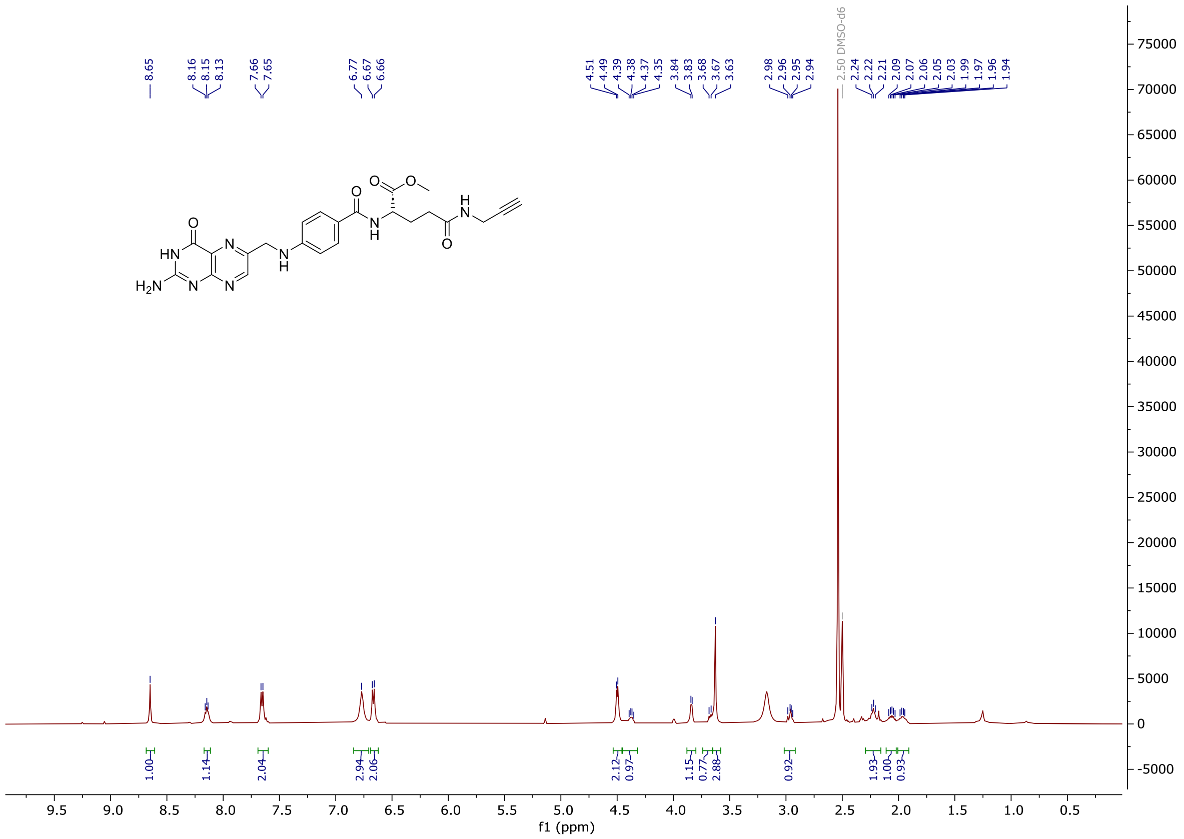

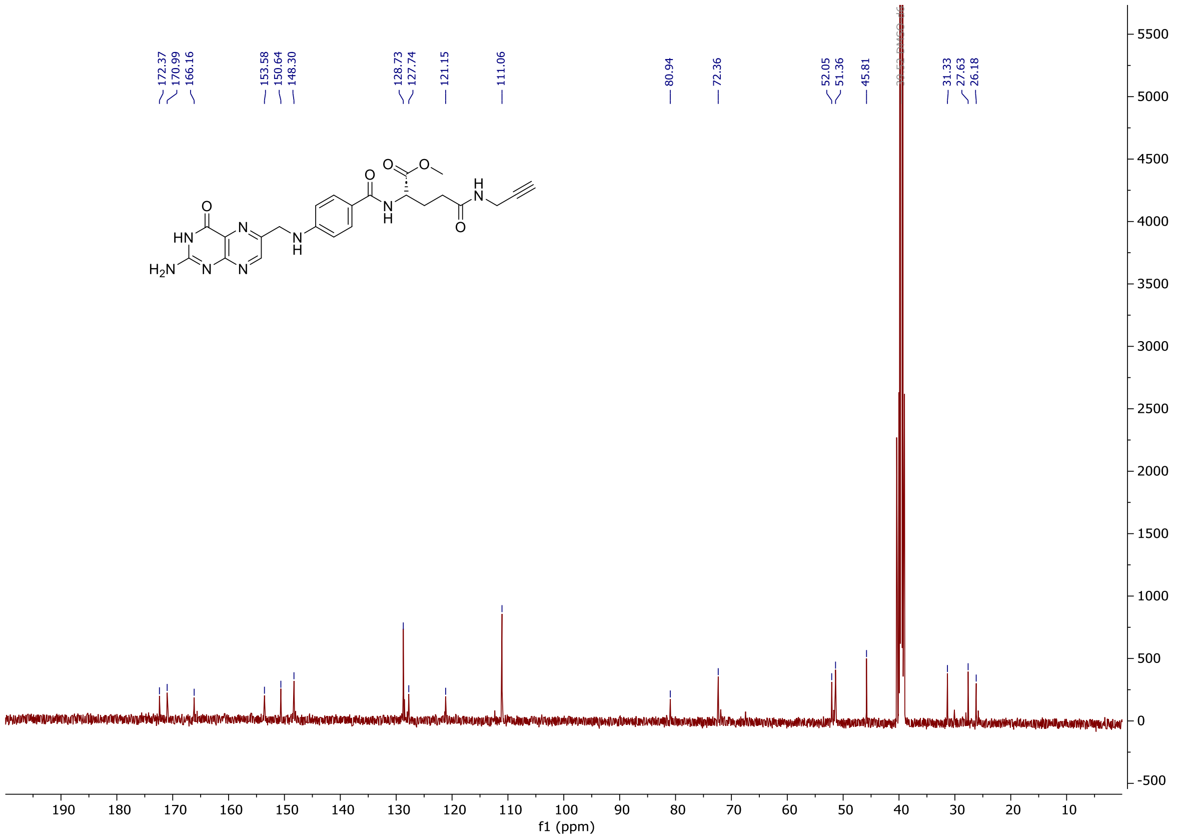

6a-Fol (Compound **8a**) – ^{1}H NMR (500 MHz), ^{13}C NMR (126 MHz) in DMSO-d$_6$

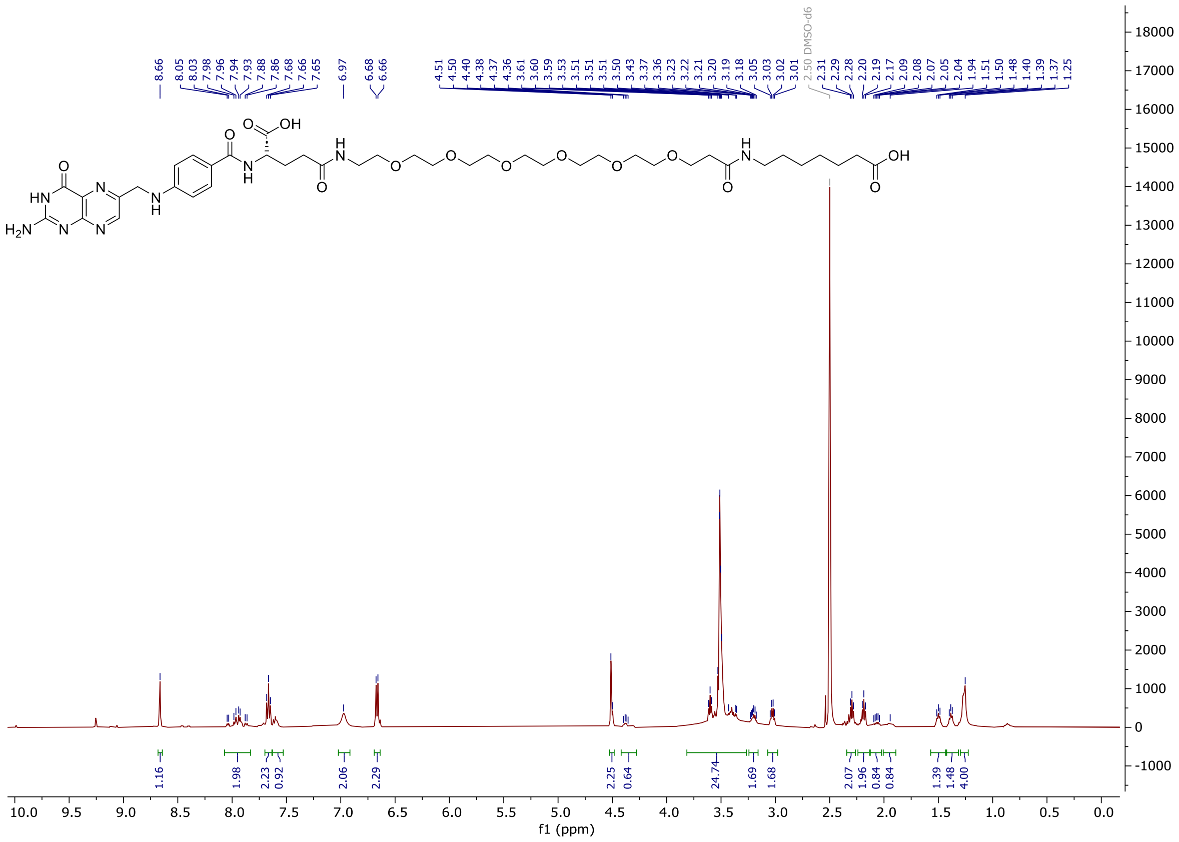

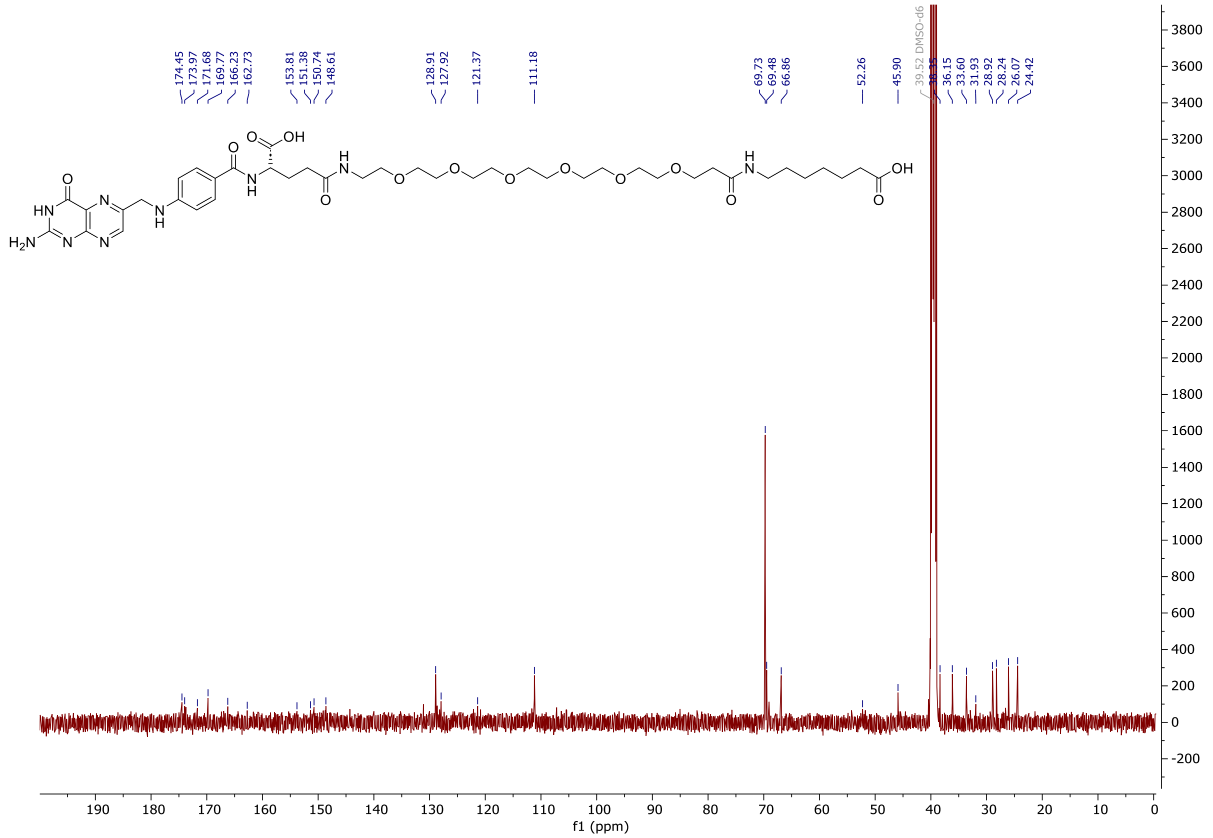

6b-Fol (Compound **8b**) – ^{1}H NMR (500 MHz), ^{13}C NMR (126 MHz) in DMSO-d_6

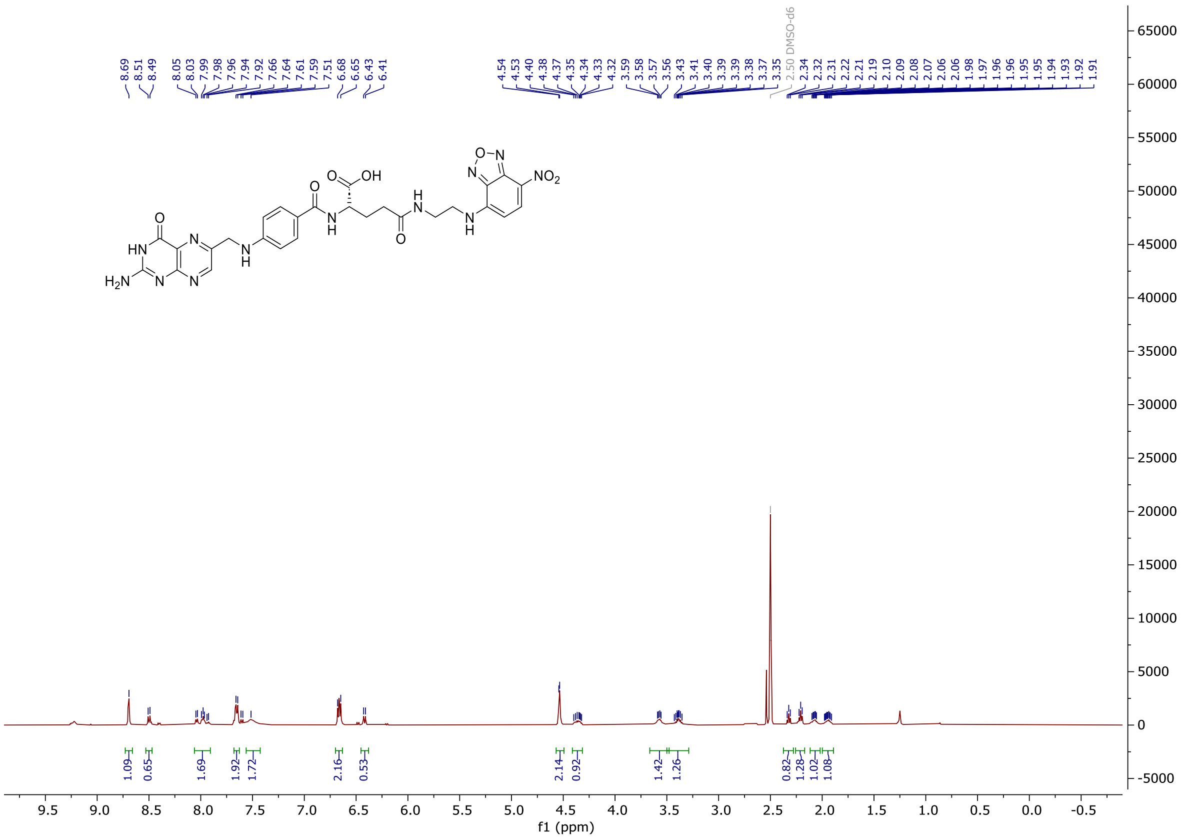

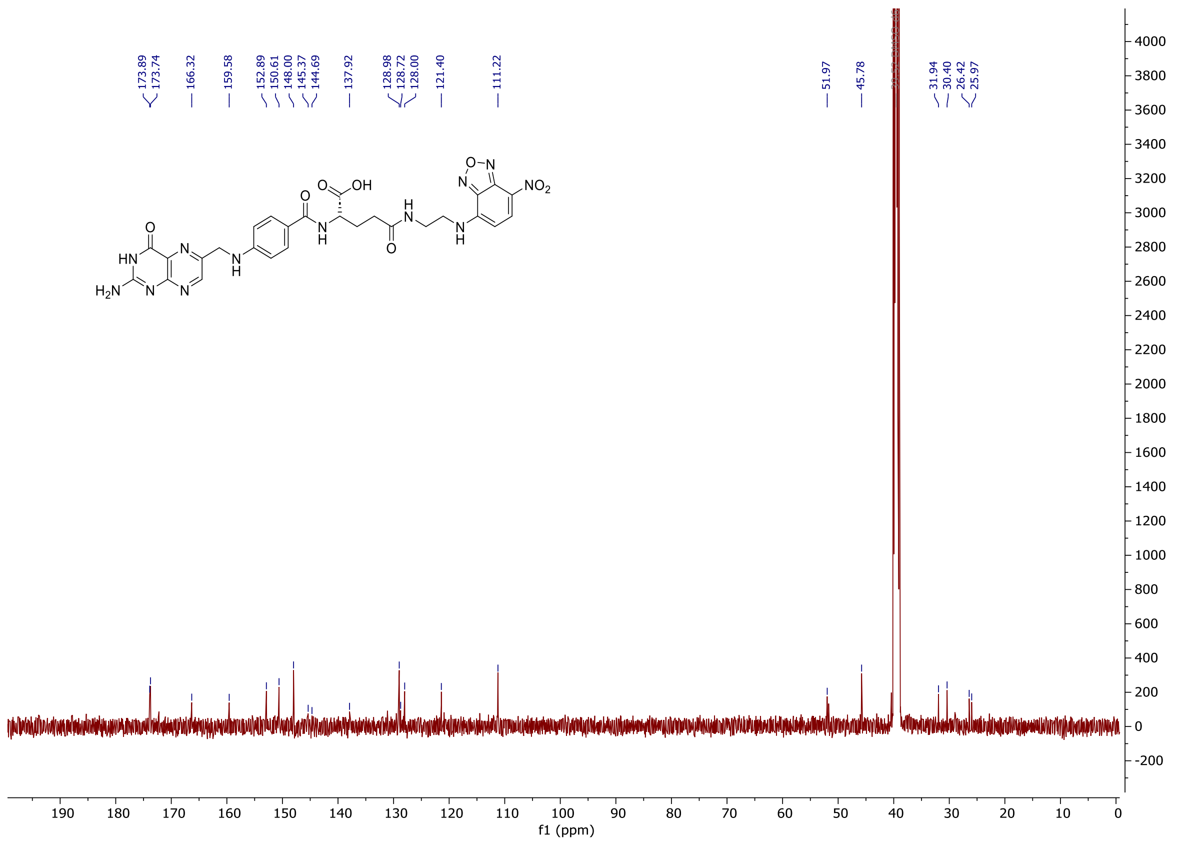

Yne-Fol (Compound **8c**) – ^{1}H NMR (500 MHz), ^{13}C NMR (126 MHz) in DMSO-d_6

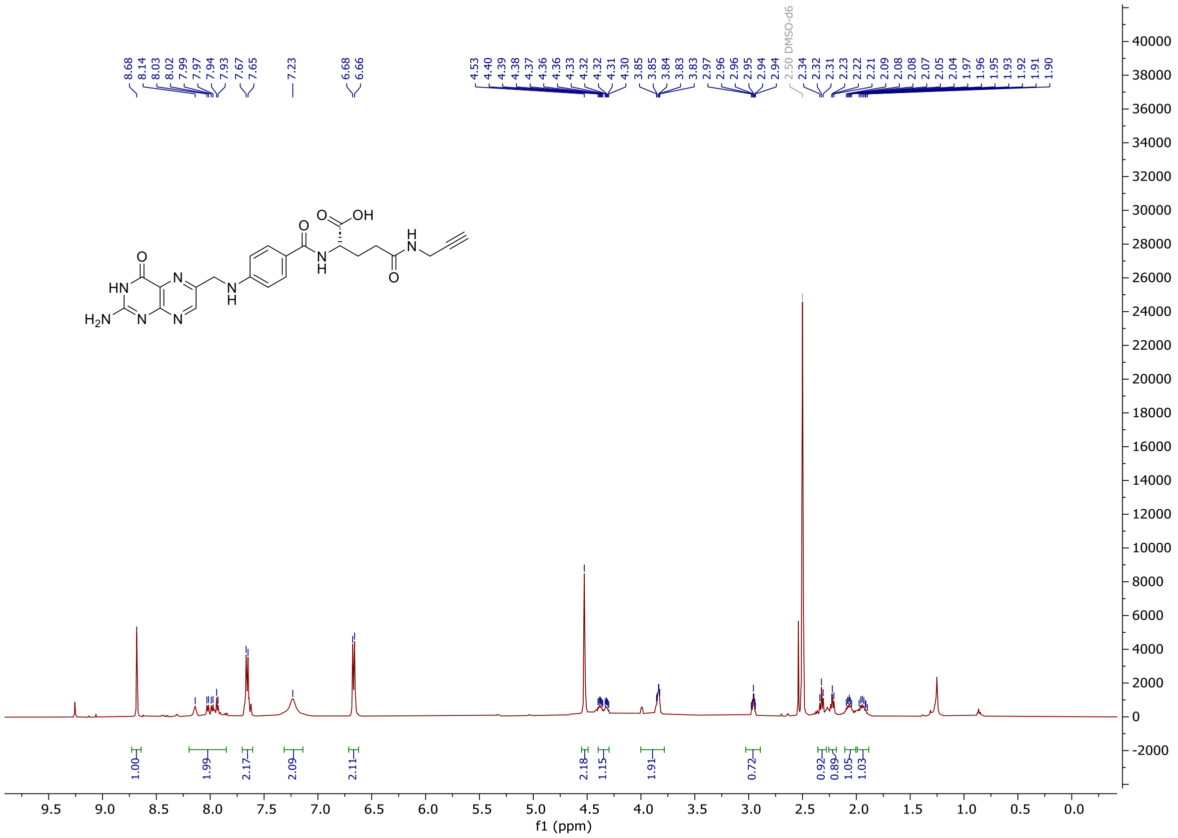

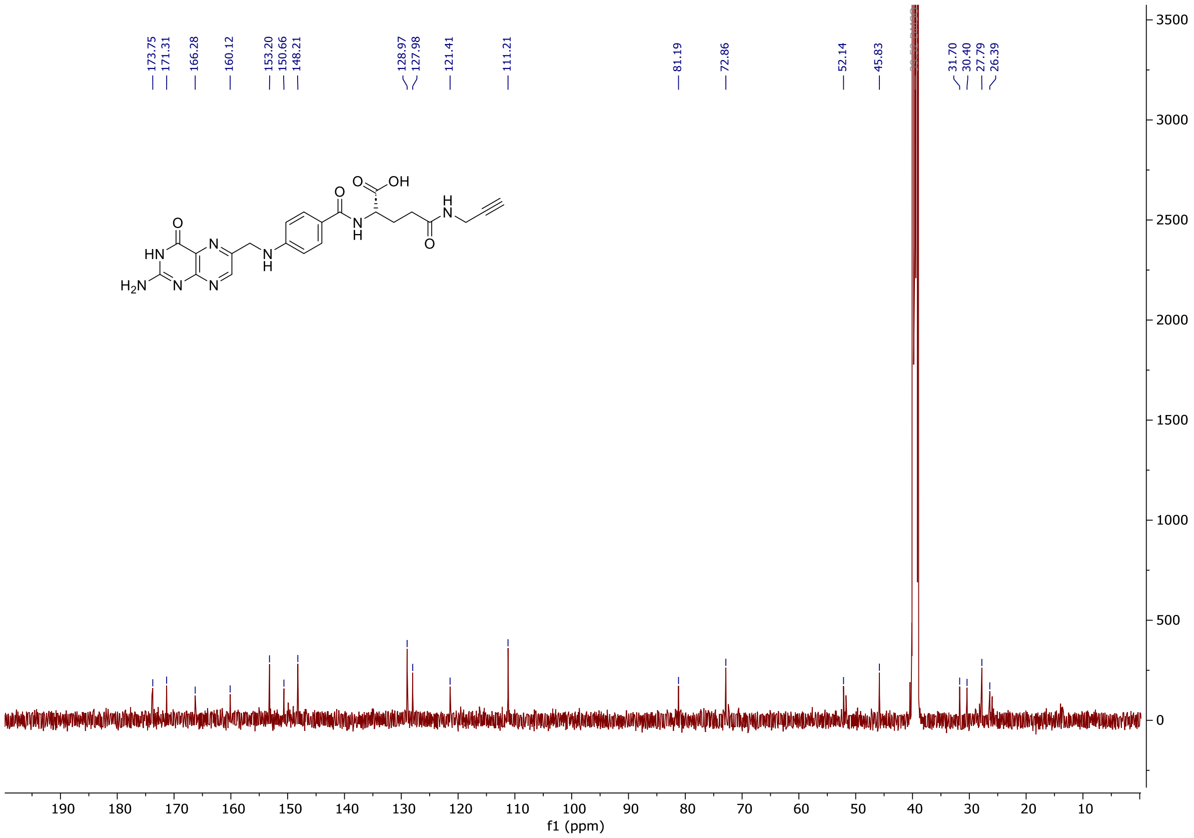

Pteroyl-L-Glu(tBu) (Compound **9**) – ^{1}H NMR (500 MHz), ^{13}C NMR (126 MHz) in DMSO-d$_6$

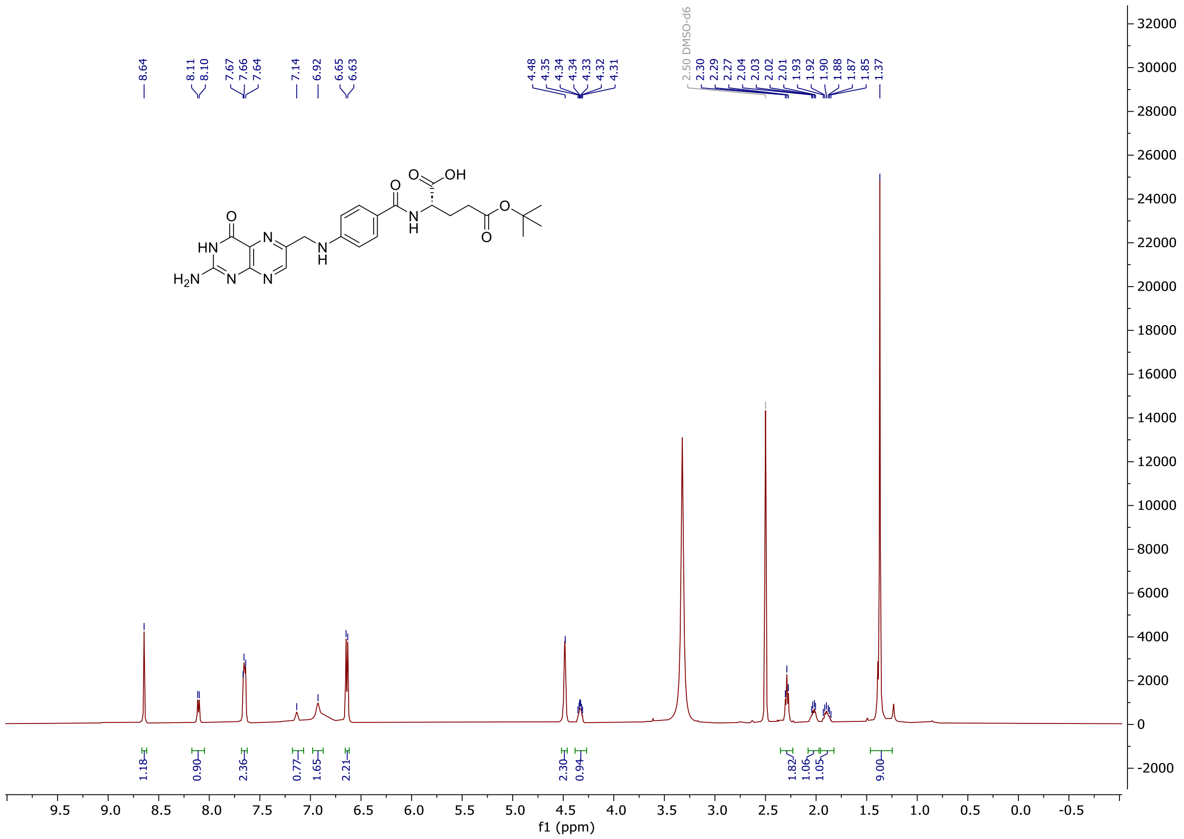

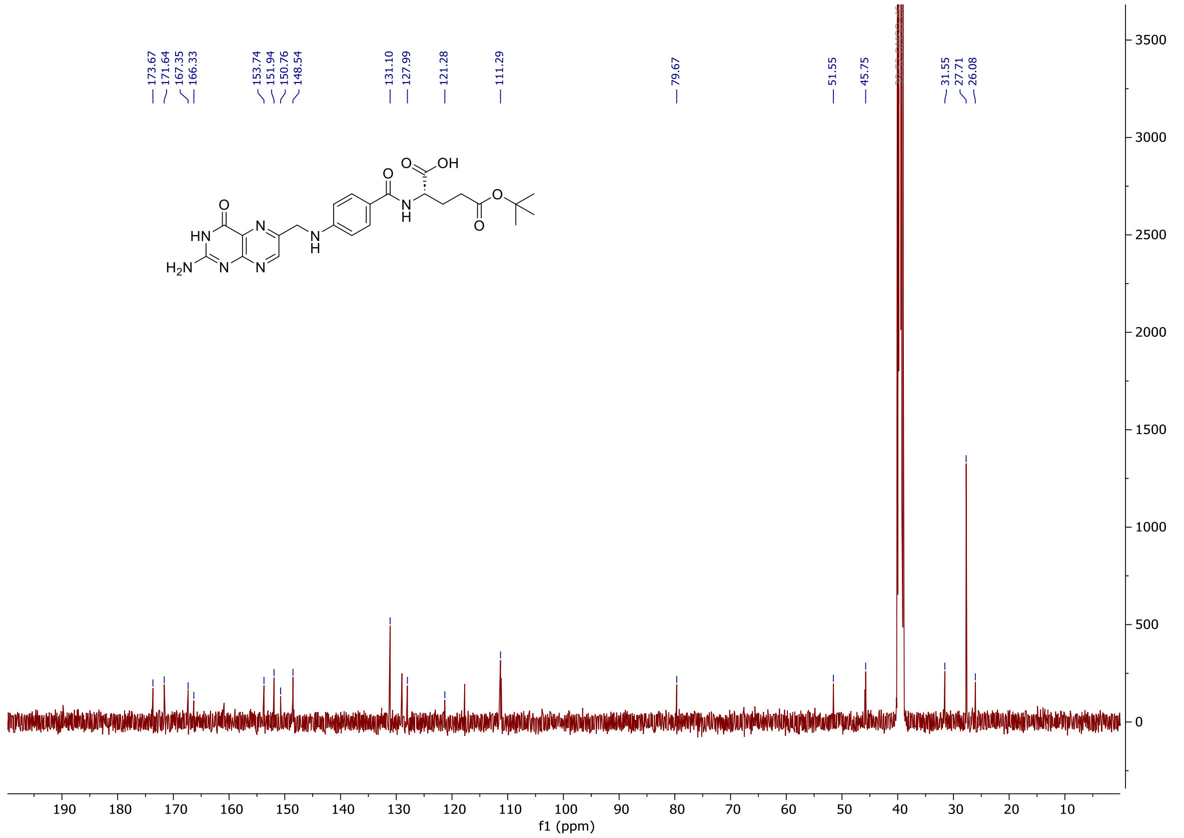

6a(OMe)-Fol (Compound **8d**) – ^{1}H NMR (500 MHz) in DMSO-d_6

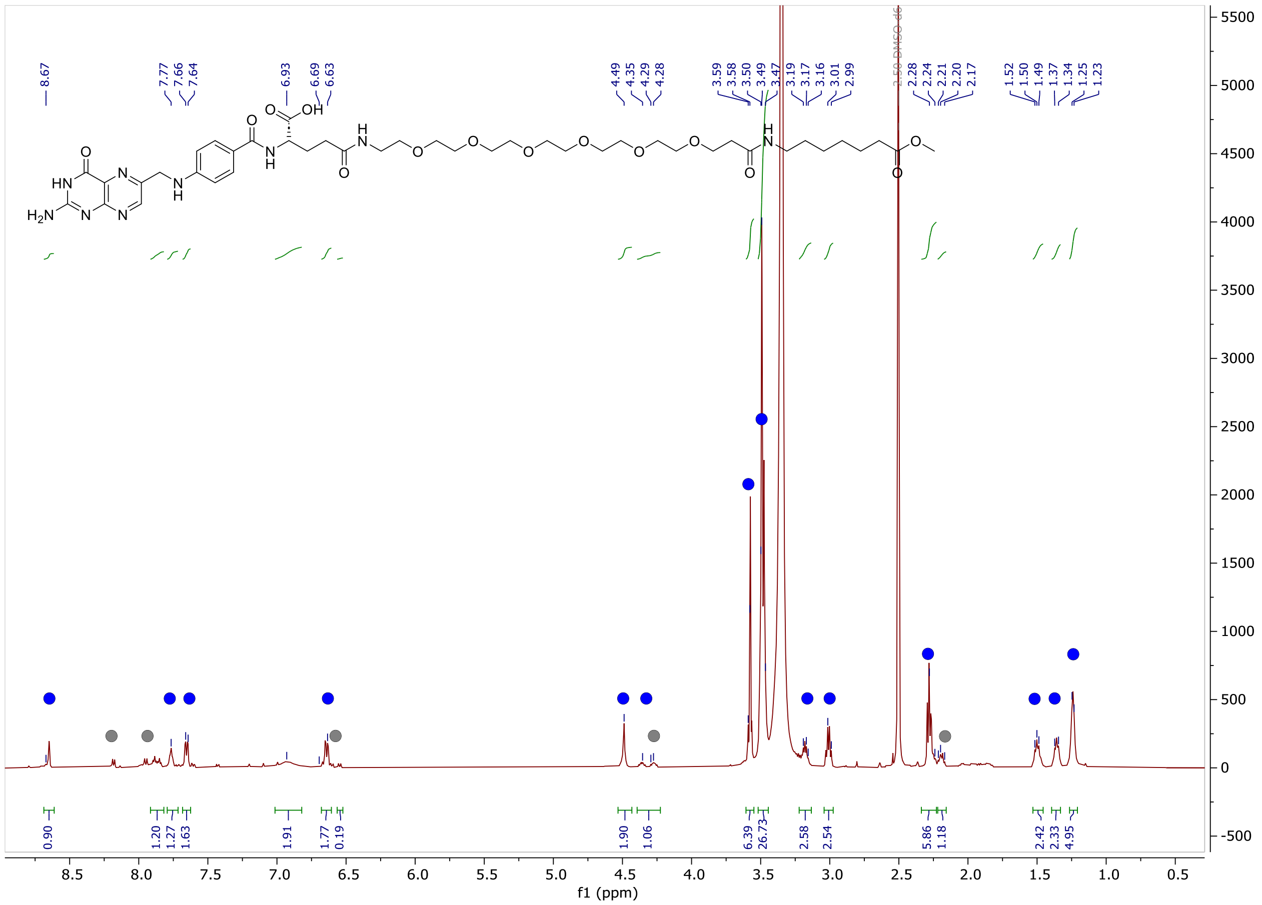

Note: Compound **8d** (blue dots) in the presence of impurities (grey dots); refer to Entry 3, Table 1.

S4. Screening of Optimal Coupling Conditions of 2 and 3 to Access 4.

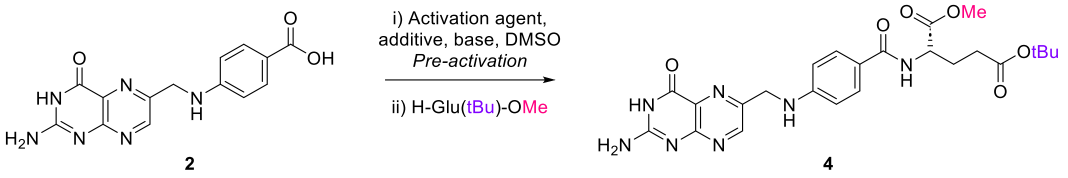

DCC vs EDC (Refer to Table 2, Entries 1-2)

DCC/HOBt/NMM(1.2 eq)

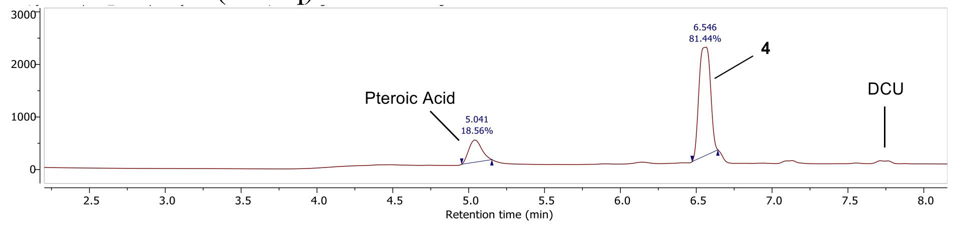

EDC/HOBt/NMM(1.2 eq)

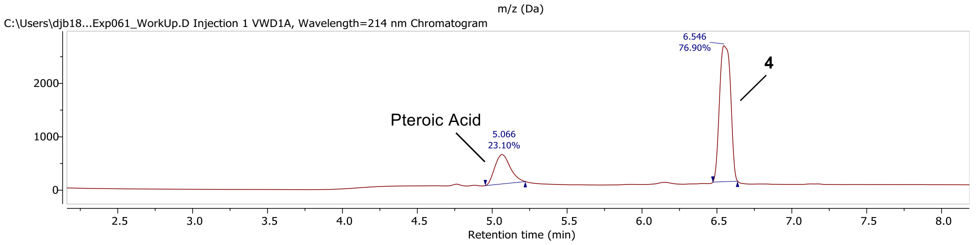

Figure S1. LC-MS analysis of crude reaction mixtures: DCC vs EDC activation reagent.

HOBt vs NHS; varying pre-activation time (Refer to Table 2, Entries 3-8)

EDC/HOBt/NMM(4 eq); 1h activation

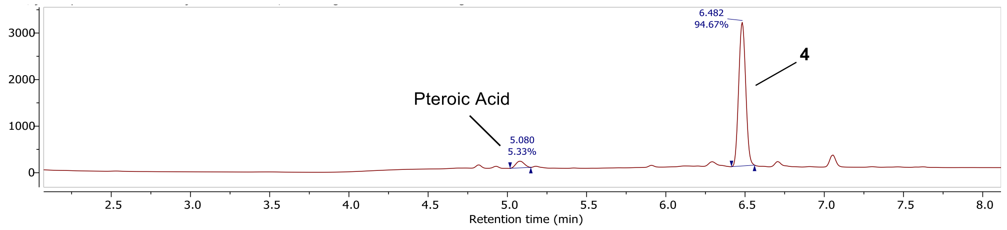

EDC/HOBt/NMM(4 eq); 4h activation

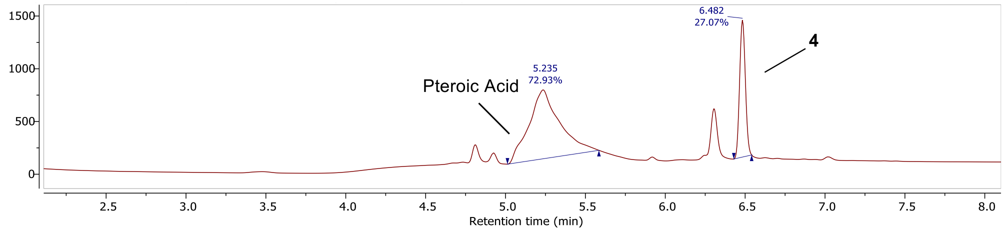

EDC/HOBt/NMM(4 eq); 6h activation

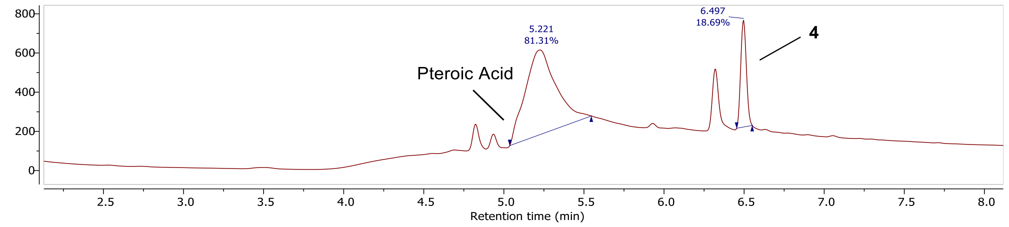

EDC/NHS/NMM(4 eq); 1h activation

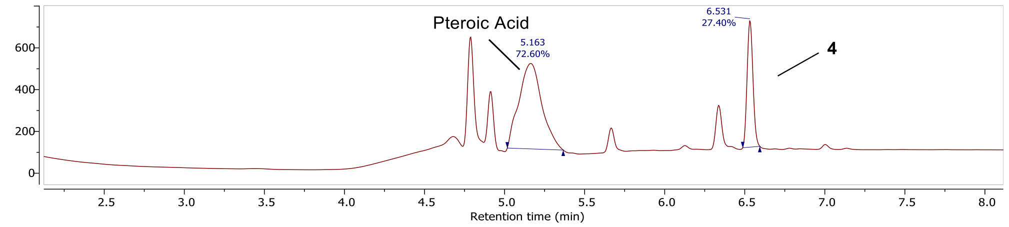

EDC/NHS/NMM(4 eq); 4h activation

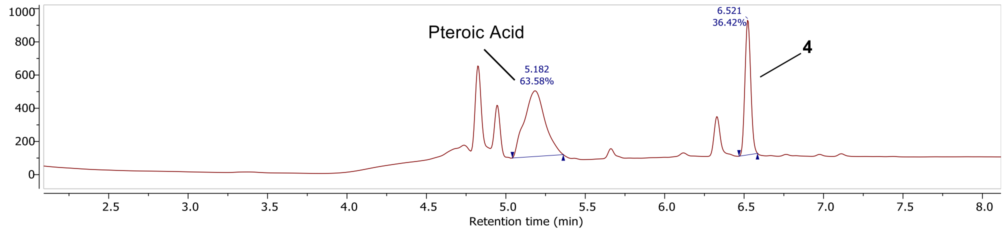

EDC/NHS/NMM(4 eq); 6h activation

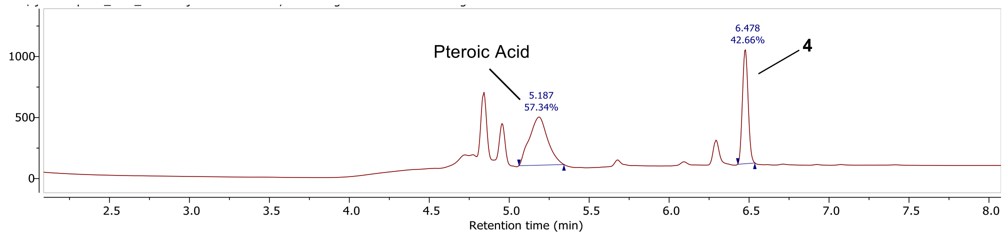

Figure S2. LC-MS analysis of crude reaction mixtures: HOBt vs NHS additive; varying pre-activation time (1, 4, or 6 h).

S5. Direct Approach vs Orthogonal Protection Route for Regioselective Conjugation of Exemplar Ligand 6a.

Scheme S1. Direct vs orthogonal route towards γ-conjugation

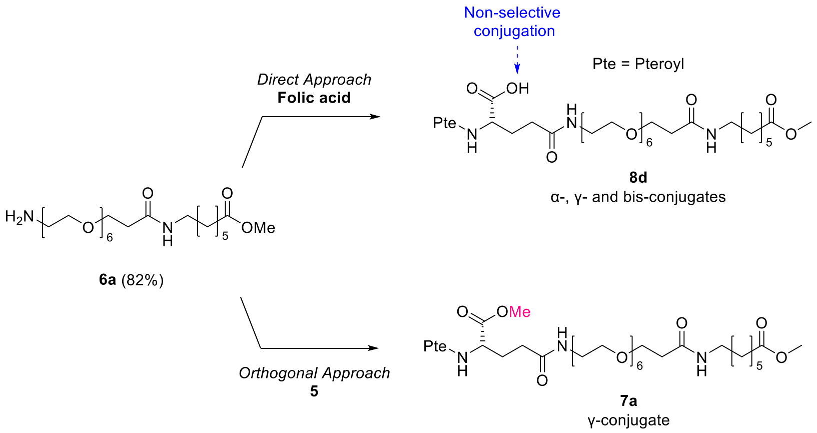

(i) Direct coupling of Folic acid with **6a** to give regioisomeric **8d**

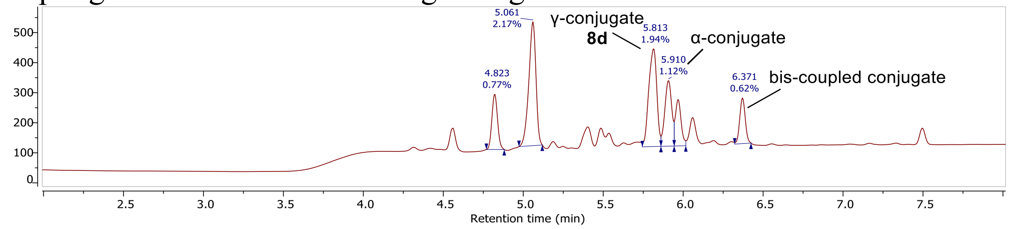

(ii) Orthogonal coupling of protected folic acid **5** with **6a** to give single γ-regioisomer **7a**

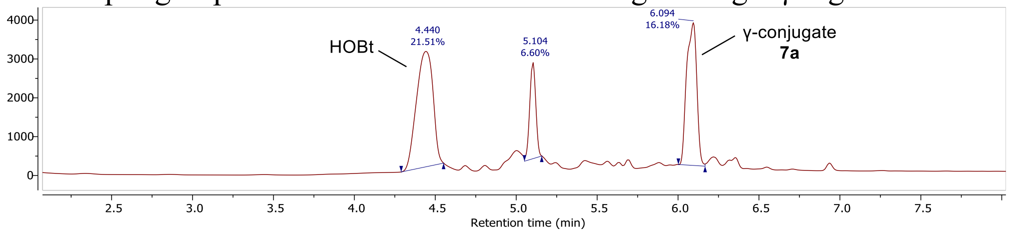

Figure S3. LC-MS analysis of crude reaction mixtures from direct vs orthogonal routes

S6. HRMS Spectra of Compounds

Pteroyl-L-Glu(tBu)-OMe (Compound **4**)
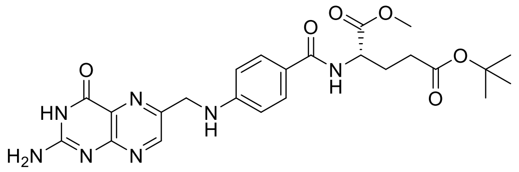 **4**
**HMRS** (ESI) *m/z*: $[M + H]^+$ Calcd for $C_{24}H_{30}O_6N_7$ 512.2252; Found 512.2241.
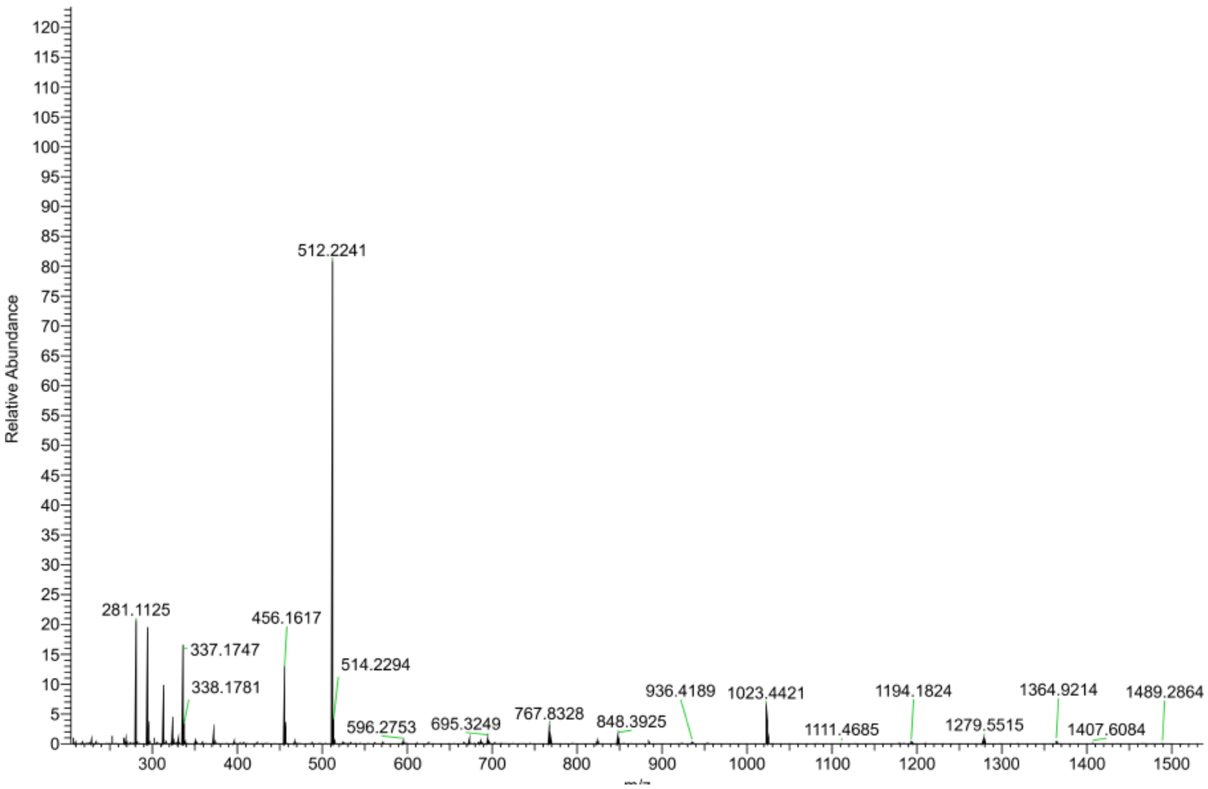

Pteroyl-L-Glu-OMe (Compound **5**)

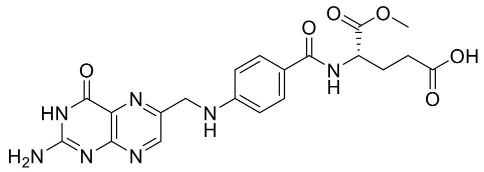

5

HMRS (ESI) *m/z*: $[M + H]^+$ Calcd for $C_{20}H_{22}O_6N_7$ 456.1626; Found 456.1614.

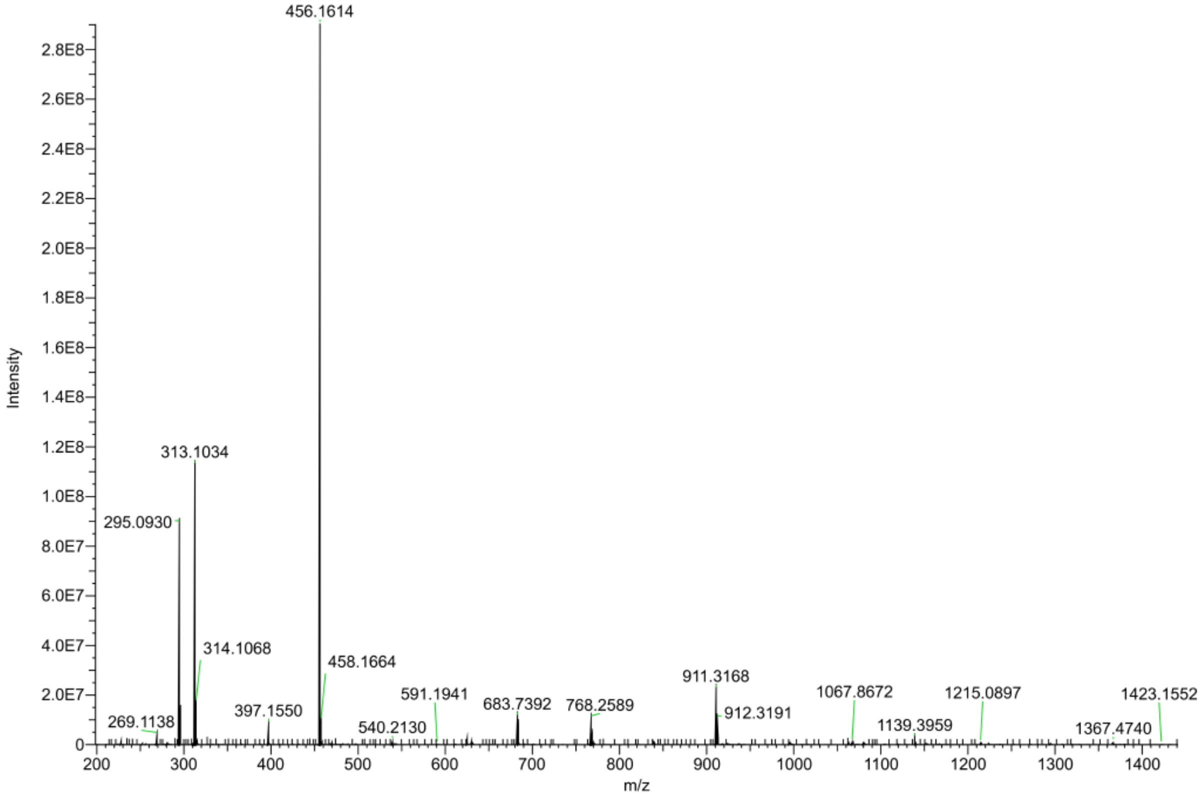

Boc-NH-(PEG)$_6$-[Alk-6]-COOMe
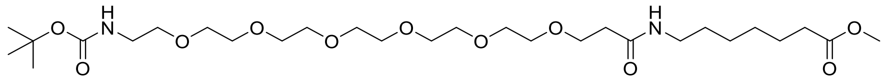
**HMRS** (ESI) *m/z*: $[M + H]^+$ Calcd for $C_{28}H_{55}O_{11}N_2$ 595.3800; Found 595.3799.
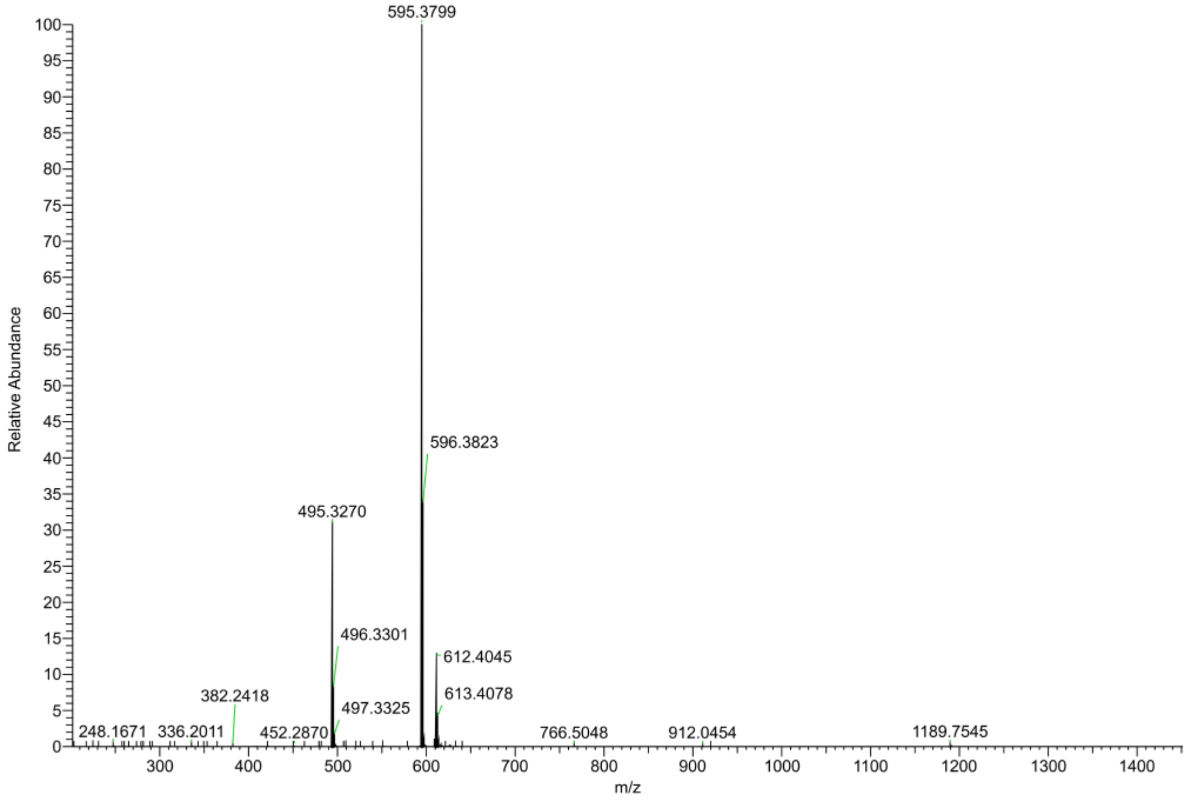

NH$_2$-(PEG)$_6$-[Alk-6]-COOMe (Compound **6a**)

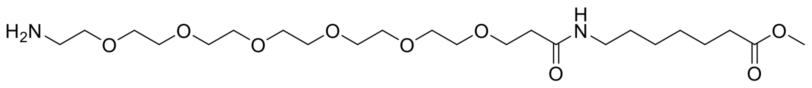

6a

HMRS (ESI) *m/z*: $[M + H]^+$ Calcd for $C_{23}H_{47}O_9N_2$ 495.3276; Found 495.3272.

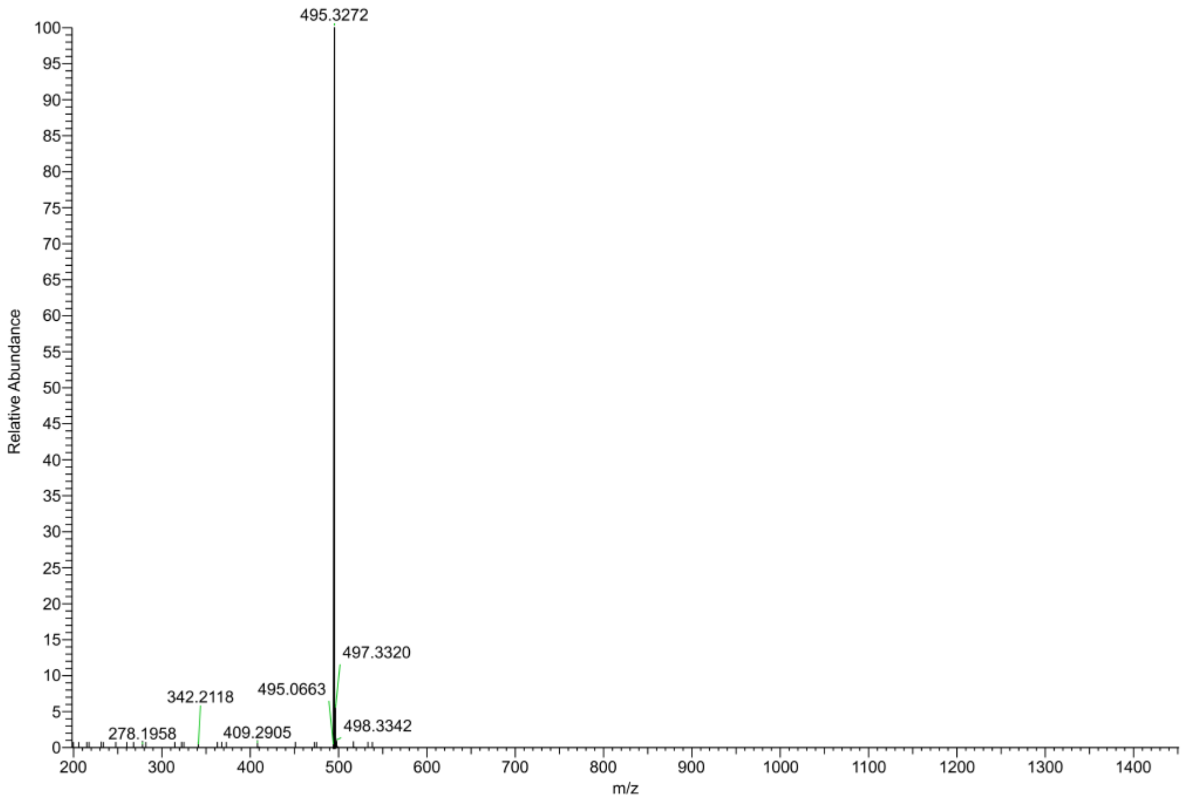

6a-Fol-OMe (Compound **7a**)

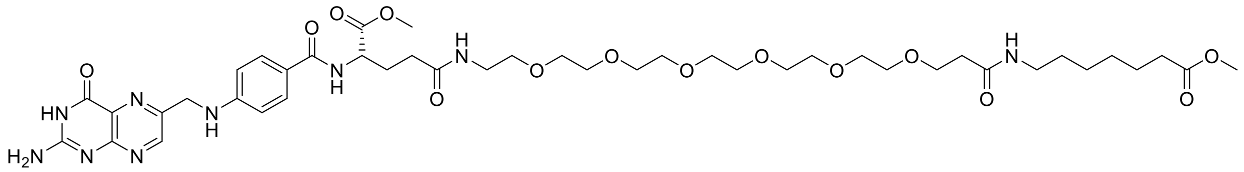

7a

HMRS (ESI) *m/z*: [M + H]$^+$ Calcd for $C_{43}H_{66}O_{14}N_9$ 932.4724; Found 932.4729.

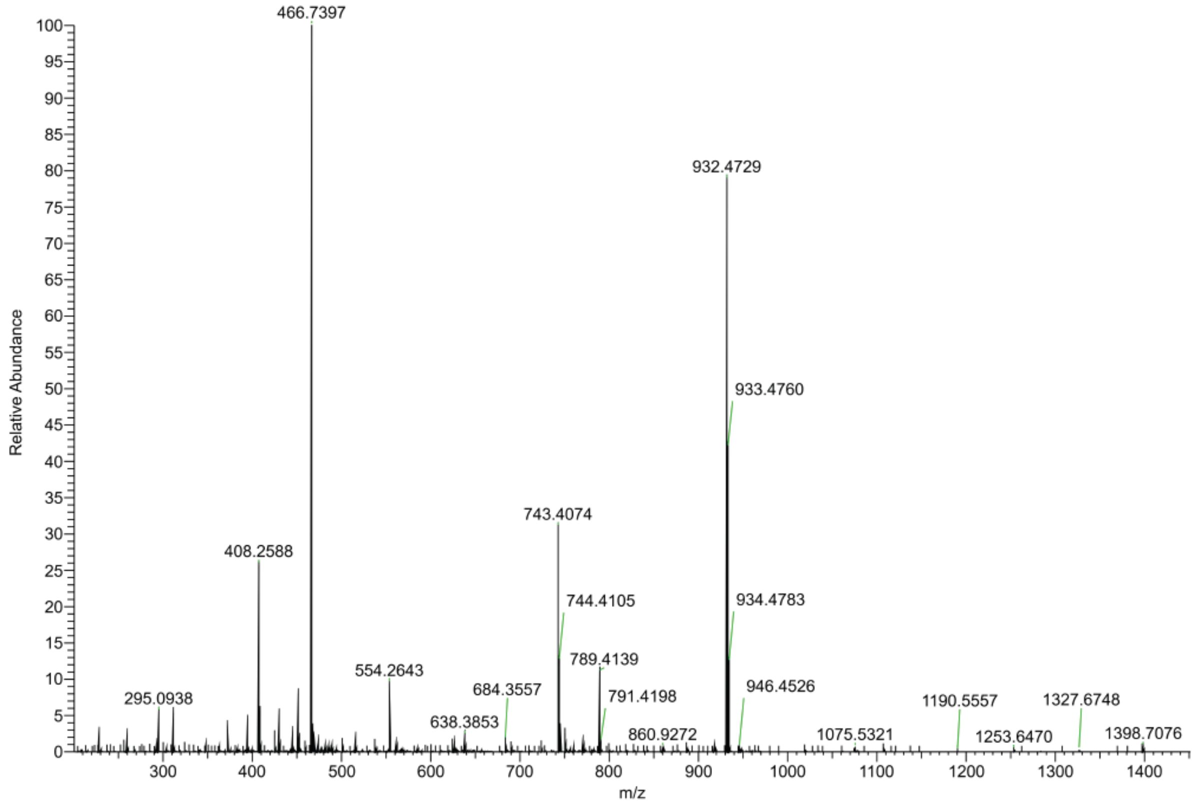

6b-Fol-OMe (Compound **7b**)
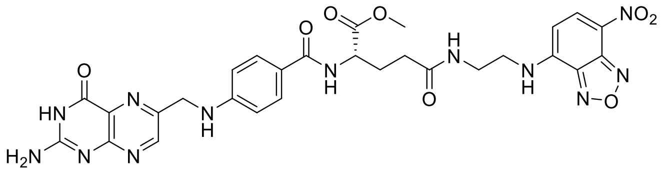 **7b**
**HMRS** (ESI) *m/z*: $[M + H]^+$ Calcd for $C_{28}H_{29}O_8N_{12}$ 661.2226; Found 661.2219.
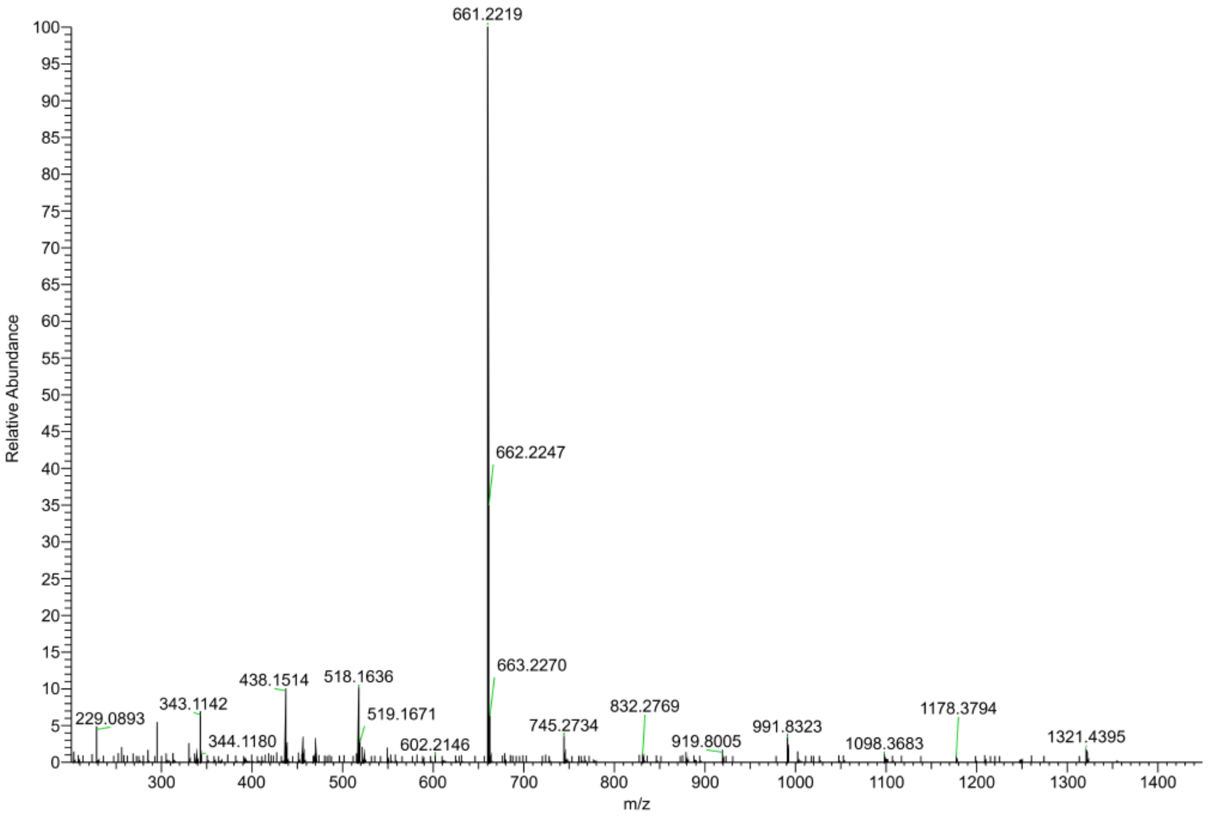

Yne-Fol-OMe (Compound **7c**)

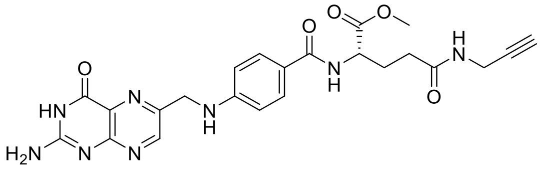

7c

HMRS (ESI) *m/z*: $[M + H]^+$ Calcd for $C_{23}H_{25}O_5N_8$ 493.1942; Found 493.1941.

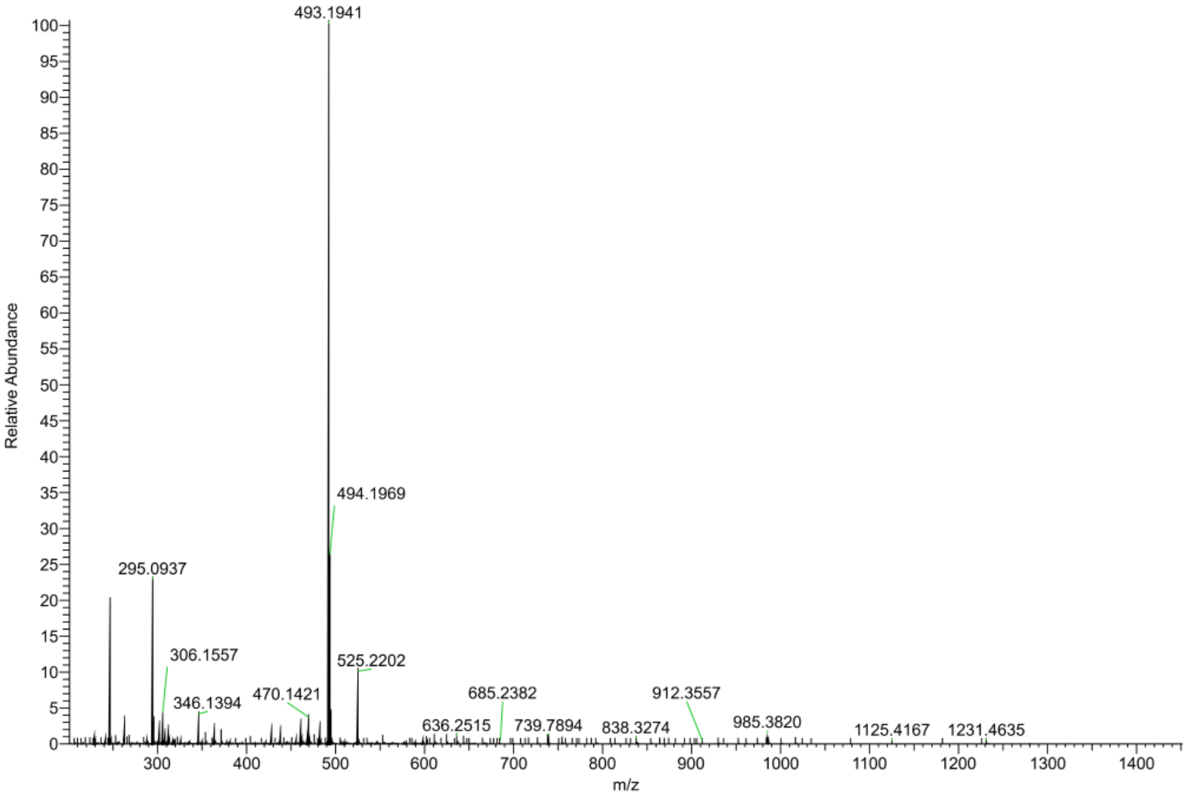

6a-Fol (Compound **8a**)
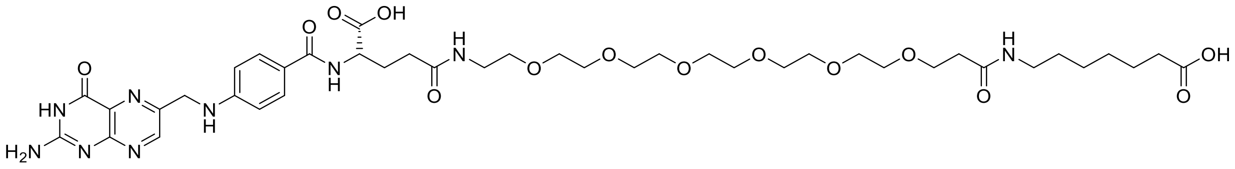 8a
**HMRS** (ESI) *m/z*: $[M + H]^+$ Calcd for $C_{41}H_{62}O_{14}N_9$ 904.4411; Found 904.4400.
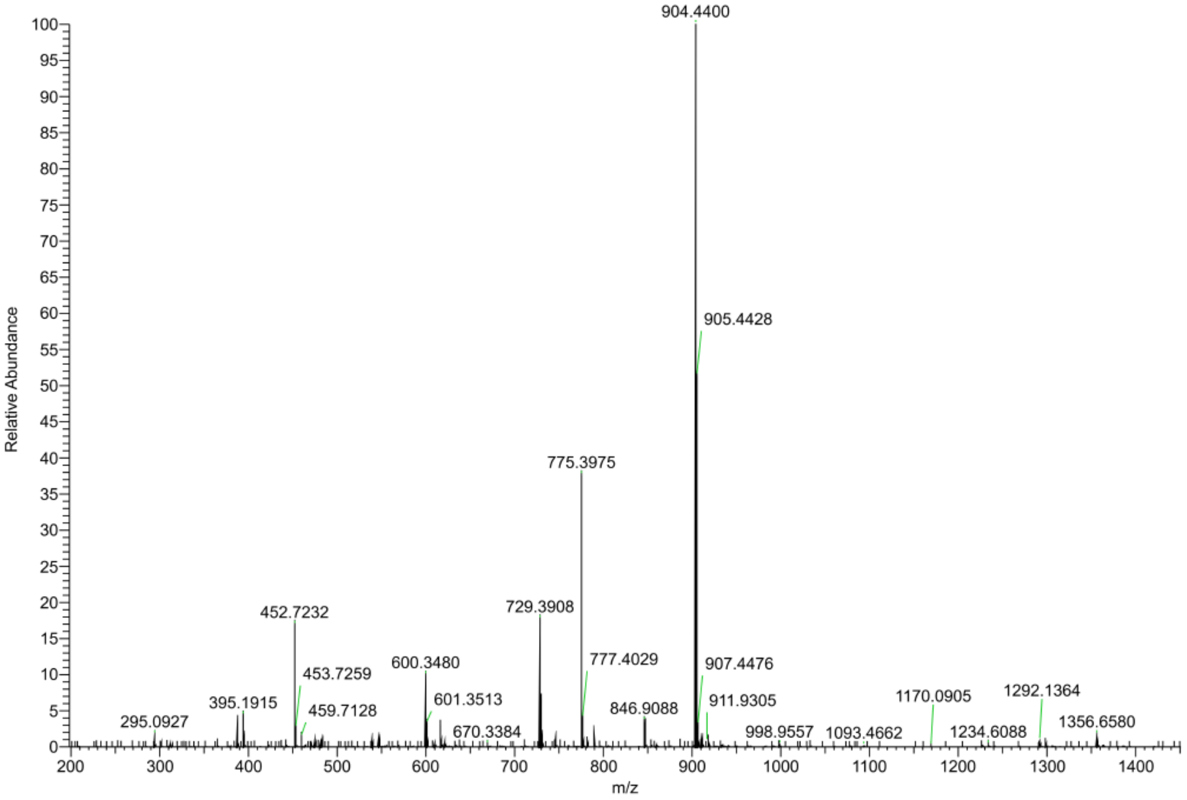

6b-Fol (Compound **8b**)

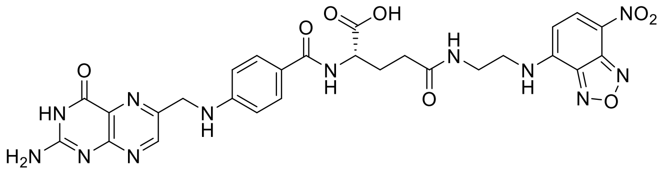

8b

HMRS (ESI) *m/z*: $[M + H]^+$ Calcd for $C_{27}H_{27}O_8N_{12}$ 647.2069; Found 647.2064.

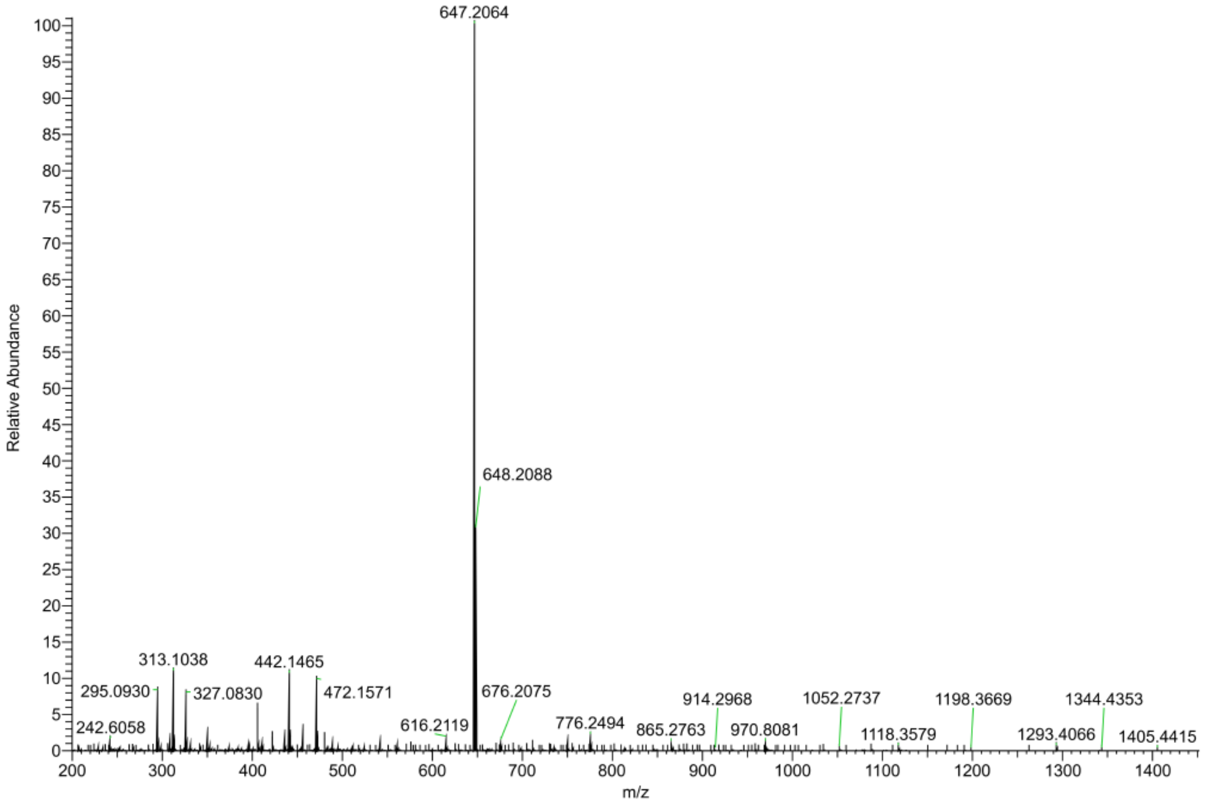

Yne-Fol (Compound **8c**)

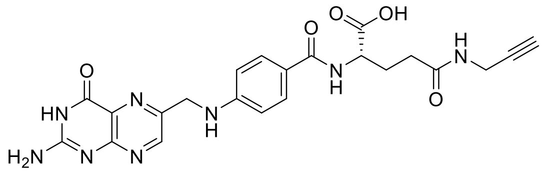

8c

HMRS (ESI) *m/z*: $[M + H]^+$ Calcd for $C_{22}H_{23}O_5N_8$ 479.1786; Found 479.1780.

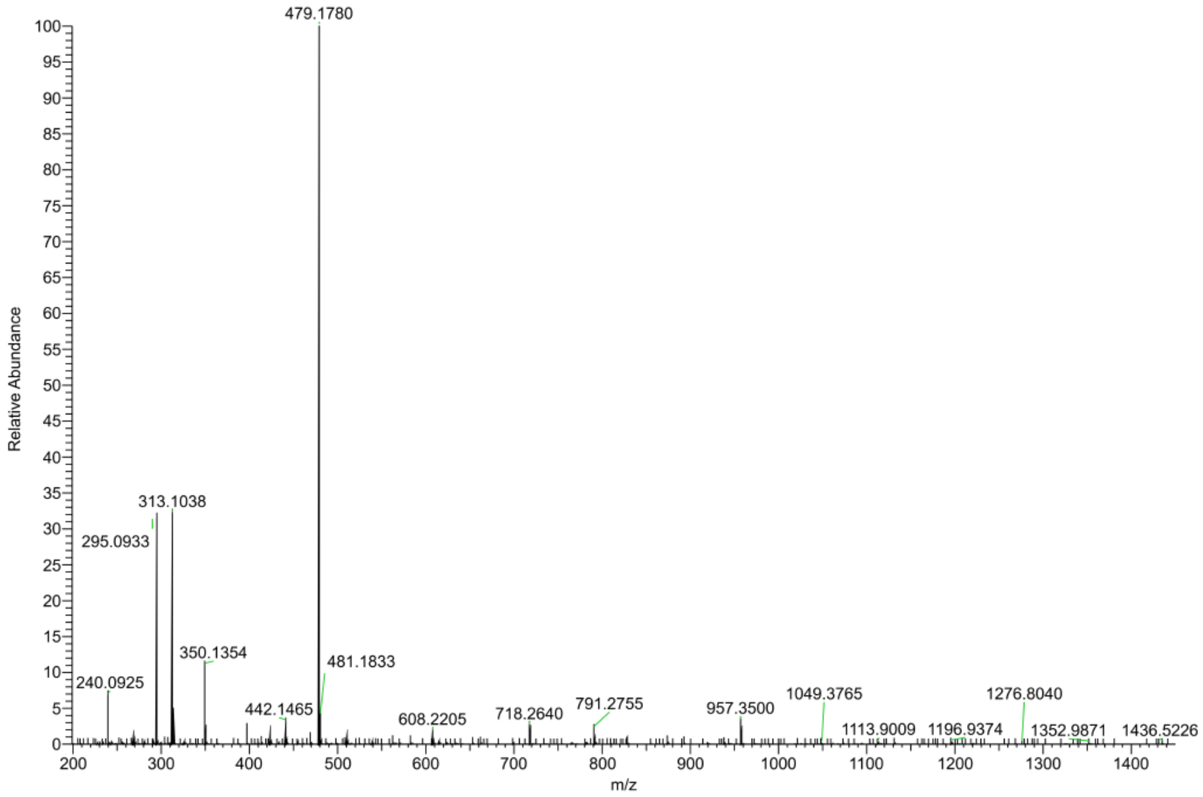

Pteroyl-L-Glu(tBu) (Compound **9**)

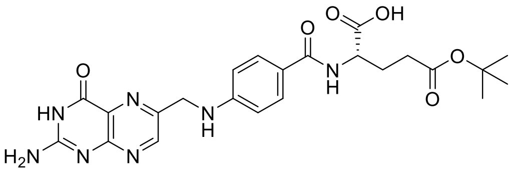

9

HMRS (ESI) *m/z*: $[M + H]^+$ Calcd for $C_{23}H_{28}O_6N_7$ 498.2096; Found 498.2086.

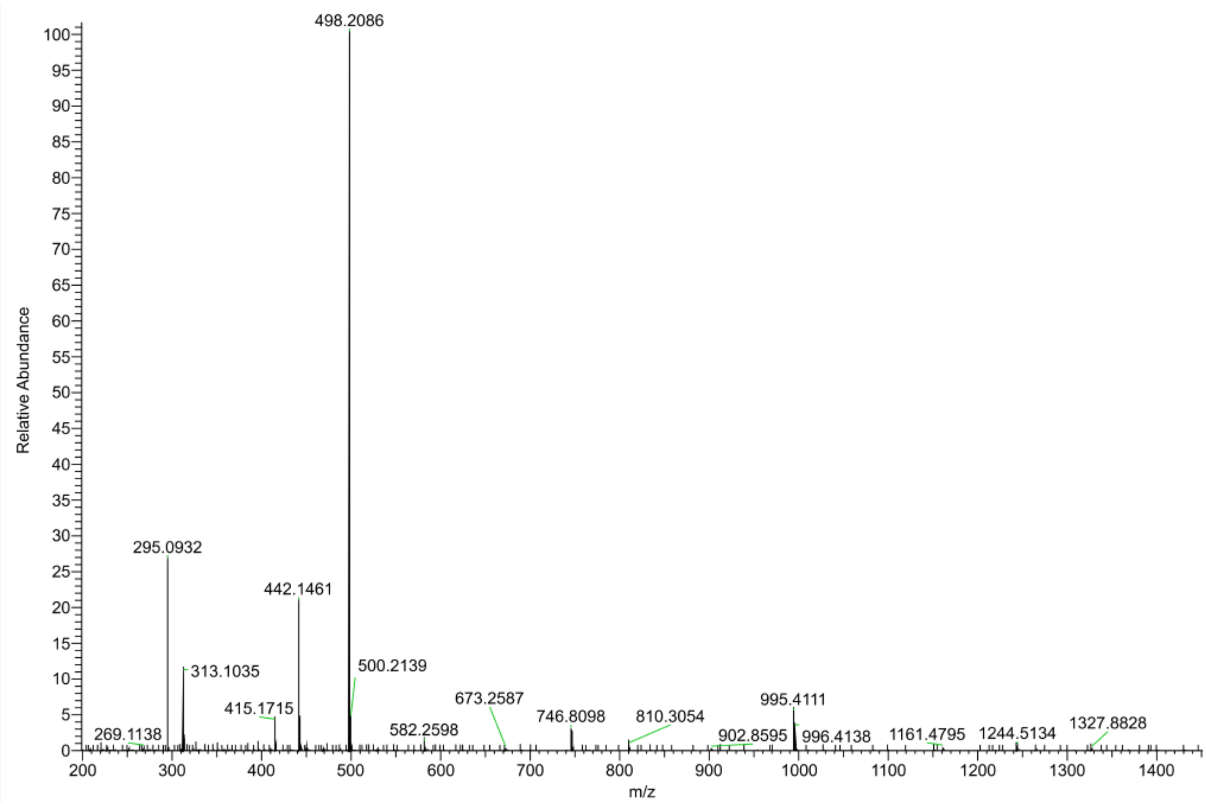

S7. References

1 R. Mazitschek, V. Patel, D. F. Wirth and J. Clardy, Development of a polarization based assay for histone deacetylase ligand discovery, *Bioorg. Med. Chem. Lett.*, 2008, **18**, 2809–2812.

2 A. Pinto, M. Wang, M. Horsman and C. N. Boddy, 6-Deoxyerythronolide B Synthase Thioesterase-Catalyzed Marcocyclization Is Highly Stereoselective, *Org. Lett*. 2012, **14**, 9, 2278–2281

3 R. Russo, R. Padanha, F. Fernandes, L. F. Veiros, F. Corzana and P. M. P. Gois, Engineering Boron Hot Spots for the Site-Selective Installation of Iminoborontes on Peptide Chains, *Chem. – Eur. J.*, 2020, **26**, 15226–15231.

4 S. Taliani, F. Simorini, V. Sergianni, C. L. Motta, F. D. Settimo, B. Cosimelli, E. Abignente, G. Greco, E. Novellino, L. Rossi, V. Gremigni, F. Spinetti, B. Chelli and C. Martini, New Fluorescent 2-Phenylindolglyoxylamide Derivatives as Probes Targeting the Peripheral-Type Benzodiazepine Receptor: Design, Synthesis, and Biological Evaluation, *J. Med. Chem*. 2007, **50**, 2, 404-407

5 (a) M. Rutnakornpituk, N. Puangsin, P. Theamdee, B. Rutnakornpituk and U. Wichai, Poly(acrylic acid)-grafted magnetic nanoparticle for conjugation with folic acid, *Polymer*, 2011, **52**, 987–995. (b) G. Baier, D. Baumann, J. M. Siebert, A. Musyanovych, V. Mailänder and K. Landfester, Suppressing Unspecific Cell Uptake for Targeted Delivery Using Hydroxyethyl Starch Nanocapsules, *Biomacromolecules*, 2012, **13**, 2704–2715

6 A. F. Trindade, R. F. M. Frade, E. M. S. Maçôas, C. Graça, C. A. B. Rodrigues, J. M. G. Martinho and C. A. M. Afonso, “Click and go“: simple and fast folic acid conjugation, *Org Biomol Chem*, 2014, **12**, 3181–3190.
